# Modulation of Morphology and Optical Property of Multi-Metallic PdAuAg and PdAg Alloy Nanostructures

**DOI:** 10.1186/s11671-018-2551-0

**Published:** 2018-05-16

**Authors:** Puran Pandey, Sundar Kunwar, Mao Sui, Sushil Bastola, Jihoon Lee

**Affiliations:** College of Electronics and Information, Kwangwoon University, Nowon-gu, Seoul, 139-701 South Korea

**Keywords:** PdAuAg nanostructures, PdAg nanostructures, Alloy nanostructures, Multi-metallic nanostructures, Solid-state dewetting, Atomic diffusion process, Plasmonic

## Abstract

In this work, the evolution of PdAg and PdAuAg alloy nanostructures is demonstrated on sapphire (0001) via the solid-state dewetting of multi-metallic thin films. Various surface configurations, size, and arrangements of bi- and tri-metallic alloy nanostructures are fabricated as a function of annealing temperature, annealing duration, film thickness, and deposition arrangements such as bi-layers (Pd/Ag), tri-layers (Pd/Au/Ag), and multi-layers (Pd/Au/Ag × 5). Specifically, the tri-layers film shows the gradual evolution of over-grown NPs, voids, wiggly nanostructures, and isolated PdAuAg alloy nanoparticles (NPs) along with the increased annealing temperature. In contrast, the multi-layers film with same thickness show the enhanced dewetting rate, which results in the formation of voids at relatively lower temperature, wider spacing, and structural regularity of alloy NPs at higher temperature. The dewetting enhancement is attributed to the increased number of interfaces and reduced individual layer thickness, which aid the inter-diffusion process at the initial stage. In addition, the time evolution of the Pd_150 nm_/Ag_80 nm_ bi-layer films at constant temperature show the wiggly-connected and isolated PdAg alloy NPs. The overall evolution of alloy NPs is discussed based on the solid-state dewetting mechanism in conjunction with the diffusion, inter-diffusion, alloying, sublimation, Rayleigh instability, and surface energy minimization. Depending upon their surface morphologies, the bi- and tri-metallic alloy nanostructures exhibit the dynamic reflectance spectra, which show the formation of dipolar (above 700 nm) and quadrupolar resonance peaks (~ 380 nm) and wide dips in the visible region as correlated to the localized surface plasmon resonance (LSPR) effect. An absorption dip is readily shifted from ~ 510 to ~ 475 nm along with the decreased average size of alloy nanostructures.

**Electronic supplementary material:**

The online version of this article (10.1186/s11671-018-2551-0) contains supplementary material, which is available to authorized users.

## Background

The recent growing interest in the development of nanodevice and applications is mainly focused on the technique to produce and design the multi-metallic nanostructures, semiconducting polymer as well as thermal transport of metal/semiconductor nanomembrane [1–10]. Multi-metallic nanostructures are essential components in various applications owing to their multi-functionality, electronic heterogeneity, and site-specific response. Multi-metallic nanostructures can add promising potentials to the development of various sensing, photovoltaic, biomedical, and catalysis applications due to the collective optical, electronic, and catalytic properties [1–6]. In specific, the multi-metallic nanostructures can offer multi-functionality, specific site response, and electronic heterogeneity, which cannot be exhibited by the monometallic counterparts [11–14]. For instance, an enhanced light absorption was demonstrated by the bimetallic Ag-Au alloy nanoclusters through the expansion of LSPR bandwidth, which resulted in the significantly improved power conversion efficiency of photovoltaics as compared to the monometallic Ag or Au nanoclusters [15, 16]. In addition, a much higher electro-catalytic activity in the electrochemical oxidation of ethanol was exhibited by the NiAuPt alloy NPs due to the synergetic effect of tri-metallic components of NPs, in which the Pt facilities the ethanol dehydrogenation while the Ni and Au remove the adsorbed intermediates simultaneously [17]. Among the various metallic elements, the Au and Ag NPs have demonstrated promising plasmonic properties while the Pd NPs have exhibited enhanced catalytic properties and chemical stability [18–20]. Therefore, the controlled fabrication of multi-metallic PdAg and PdAuAg nanostructures by the physical deposition can find further opportunities in the related applications, which, however, has not been reported till now. In this paper, the systematic fabrication of PdAg and PdAuAg nanostructures is demonstrated through the solid-state dewetting on sapphire (0001). The growth dynamics was sharply and systematically compared by using the same thickness of 15 nm tri-layers (Pd/Au/Ag) and multi-layers (Pd/Au/Ag × 5). Various growth parameters such as annealing temperature, annealing duration, film thickness, and deposition order are systematically controlled to achieve diverse configuration, size, and density of PdAg and PdAuAg nanostructures. The evolution of nanostructures is mainly analyzed based on the inter-diffusion, alloying, and diffusion of constituent alloy atoms as well as Rayleigh instability and surface energy minimization mechanisms. The reflectance spectra of corresponding PdAuAg nanostructures exhibit the gradual evolution of absorption dip, quadrupolar, and dipolar resonance peaks at specific wavelength along with the morphology evolution. On the other hand, dynamic plasmonic behavior is observed in the reflectance spectra depending on the size evolution of nanostructures.

## Methods/Experimental

### Materials Preparation and Thin Film Deposition

**Fig. 1:**
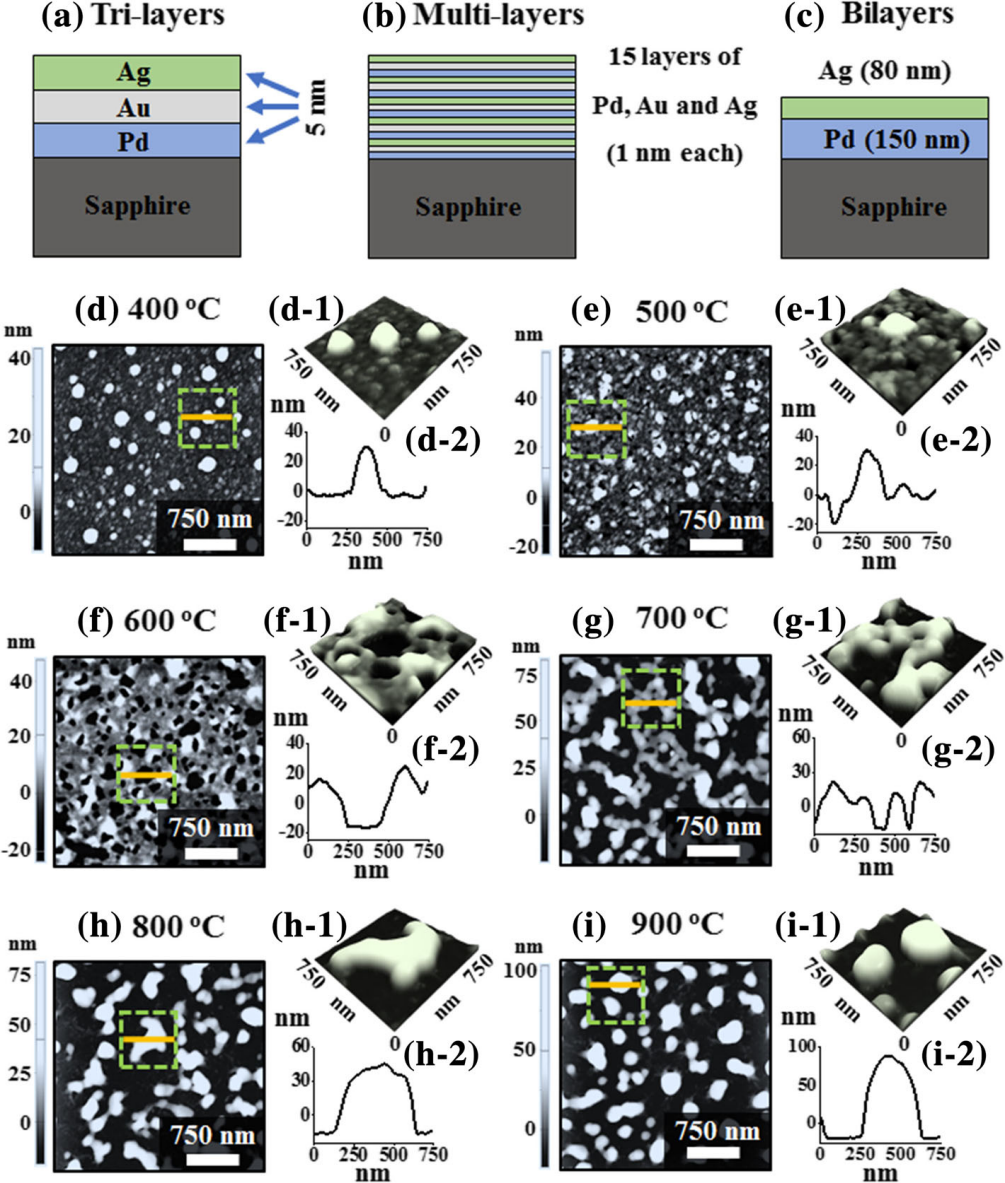
Schematic description of layer deposition. **a** 15 nm of Pd/Au/Ag with 5 nm each layer (tri-layers). **b** 15 nm of Pd/Au/Ag (15 layers) with 1 nm each (multi-layers). **c** Pd_150 nm_/Ag_80 nm_ bi-layer deposition (bi-layers). **d**–**i** Evolution of tri-metallic PdAuAg NPs between 400 and 900 °C for 450 s on sapphire (0001). This set is based on the 15 nm of Pd/Au/Ag with 5 nm each layer. **d**–**i** AFM top-views (3 × 3 μm^2^). **d-1**–**i-1** Magnified AFM side views (750 × 750 nm^2^). **d-2**–**i-2** Cross-sectional line profiles

Initially, 430-μm-thick sapphire (0001) wafers with an off-axis of ± 0.1° (iNexus Inc., South Korea) were diced into 6 × 6 mm^2^ by using a machine saw. Then, the samples were degassed at 600 °C for 30 min under 1 × 10^− 4^ Torr in a pulsed laser deposition (PLD) chamber to remove various particles and oxides. After the degassing, the substrates were atomically smooth as shown in Additional file 1: Figure S1(b). In this work, three sets of samples were prepared for the investigation of morphological and optical properties of various multi-metallic nanostructures as shown in Fig. 1a–c. Both tri-layer and multi-layer samples were consisted of 15 nm-thick tri-metallic films of Pd, Au, and Ag with the different deposition scheme. For example, the tri-layer samples were consisted of Pd (5 nm), Au (5 nm), and Ag (5 nm) as shown in Fig. 1a while the multi-layer samples contained the Pd (1 nm), Au (1 nm), and Ag (1 nm) films with 5 times repeated as shown in Fig. 1b. The bi-layer samples were depositted with the Pd_150 nm_/Ag_80 nm_ as shown in Fig. 1c. The film deposition was done by a sputter at a deposition rate of 0.1 nm/s at the 3 mA ionization current under 1 × 10^− 1^ Torr at an ambient temperature.

### Fabrication of PdAuAg and PdAg Alloy Nanostructures

After the depositions, both the tri-layer and multi-layer samples showed smoother morphologies as shown in Additional file 1: Figure S1(c)–(d). Subsequently, the tri-layer and multi-layer PdAuAg samples were annealed at various temperature between 400 and 900 °C to investigate the evolution process along with temperature control in a PLD chamber under 1 × 10^− 4^ Torr. Each target temperature was reached at a ramping rate of 4 °C/s. For the Pd/Ag bi-layer samples, the annealing duration was systematically varied between 0 and 3600 s at 850 °C to see the time behavior. The temperature was chosen to ensure a sufficient dewetting of thick Pd/Ag bi-layers.

### Characterization of PdAuAg and PdAg Alloy Nanostructures

The surface morphology was characterized by an atomic force microscope (AFM) (XE-70, Park Systems Corp., United States of America). The scanning was performed under a non-contact mode at an ambient condition. A scanning electron microscope (SEM) (COXEM, CX-200, South Korea) was utilized for the large-scale morphology characterization operated at 20 kV in vacuum. The elemental analysis and maps of samples were obtained by the energy-dispersive X-ray spectroscope (EDS) system (Thermo Fisher, Noran System 7, United States of America), operated under vacuum. The optical characterization (UV-VIS-NIR reflectance) of corresponding samples was performed using an UNIRAM II system (UniNanoTech Co. Ltd., South Korea).

## Results and Discussion

**Fig. 2:**
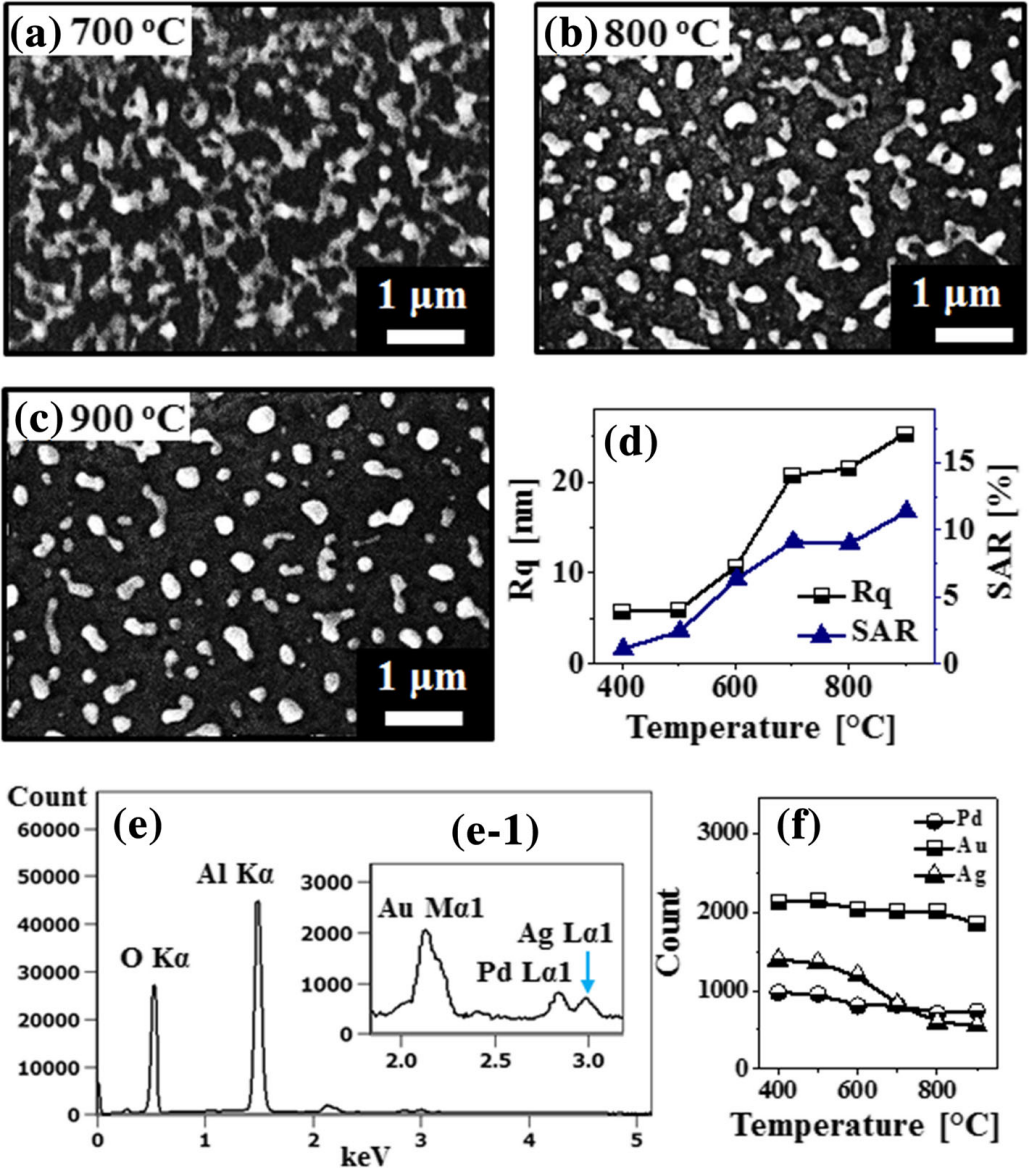
**a**–**c** SEM images of tri-metallic PdAuAg NPs annealed between 700 and 900 °C (tri-layers). **d** Plots of RMS surface roughness (Rq) and surface area ratio (SAR) of corresponding samples as a function of annealing temperature. **e** Energy dispersive X-ray spectroscopy (EDS) full spectrum of sample annealed at 400 °C. **f** Plots of Pd, Au, and Ag EDS counts

Figure 1d–i show the evolution of PdAuAg nanostructures from the Pd/Au/Ag tri-metallic layers as shown in Fig. 1a by the systematic annealing between 400 and 900 °C for 450 s. As demonstrated by the AFM top-views and side-views, distinctive surface morphologies were observed depending upon the annealing temperature such as over-grown alloy NPs, voids, wiggly nanostructures, and isolated PdAuAg alloy NPs. The overall evolution process of PdAuAg alloy NPs can be discussed based on the solid-state dewetting (SSD) of tri-layers metallic film. Generally, through the SSD, the uniform thin film transforms into the isolated particles well below the melting point, which can be induced by the surface diffusion of the atoms upon annealing. In the case of multi-metallic system, the SSD can be affected by the atomic interdiffusion and alloying of atoms. Nevertheless, the overall SSD is driven by the surface and interface energy minimization of the thermodynamic system [21, 22]. The SSD process mainly depends on the properties of constituent metallic films such as surface energies, diffusivities, interface energies, and growth parameters such as temperature, duration, pressure, and initial film thickness. In this case, the metallic tri-layers consists of three miscible components Pd, Au, and Ag, having same fcc crystal structure with slightly different lattice constant [23]. The Pd, Au, and Ag films have different surface energies and atomic diffusivities. In specific, the surface energies of Pd, Au, and Ag are 1808, 1363, and 1065 mJ/m^2^ respectively [24]. Thus, the surface diffusivity of Ag atoms is highest and then the Au and Pd atoms in the order of Ag > Au > Pd. This difference in the surface diffusivity of Pd, Au, and Ag atoms can significantly affect the inter-diffusion process. Upon annealing, the atom starts to diffuse through the surface and interface of different metal layers giving rise to the inter-diffusion process. Due to the high diffusivity of Au and Ag atoms, higher inter-diffusion at the Au-Ag interface can be expected as compared to the Pd-Au interface. In the meantime, the inter-diffusion can be enhanced by increasing the annealing temperature as given by the Arrhenius relation: inter-diffusion coefficient $(D)={D}_0\ \exp \left(-\frac{E_a}{\mathrm{kT}}\right)$, where *D*_0_, *E*_*a*_, *T*, and *K* are the pre-exponential diffusivity, activation energy of inter-diffusion, temperature, and Boltzmann constant, respectively. Thus, along with the increased annealing temperature, the inter-diffusion at Au-Ag and Au-Pd interfaces can be enhanced and ultimately the Pd, Au, and Ag atoms can all inter-mix, resulting in the formation of PdAuAg alloy [21]. Subsequently, the dewetting process can be initiated with the formation of tiny pinhole and void at lower energy sites owing to the coalescence of atomic vacancies [22]. Along with the increased temperature, the diffusion of alloyed atoms can be further enhanced and as a result the void can grow bigger by the coalescence of nearby ones due to the capillary forces around void rims. These void rims eventually become unstable and evolves as a wiggly nanostructure at increased temperature due to the surface energy anisotropy. Finally, at high annealing temperature, the wiggly nanostructure can break into isolated alloy NPs due to the Rayleigh-like instability based on the minimization of surface energy [25]. In this case, initially, the over-grown NPs were observed at 400 °C as shown in Fig. 1d, d-1. These over-grown NPs can be partially alloyed AuAg NPs that can be formed due to the enhanced inter-diffusion and rapid accumulation of the Au and Ag atoms at the upper interface, i.e., Au-Ag interface [21, 24]. The typical over-grown alloy NPs had around 200 nm width and 30 nm height as extracted by the cross-sectional line profile in Fig. 1d-2. When the temperature was increased to 500 °C, the inter-diffusion between Pd, Au, and Ag atoms at Ag-Au and Au-Pd interfaces can be further enhanced and thus PdAuAg alloy system can be formed. At the same time, the atomic vacancies in the film can be coalesced, resulting in the formation void especially around the low energy sites as shown in Fig. 1e, e-1. The over-grown alloy NPs were gradually immersed into the voids, which can be correlated to the enhanced diffusion of alloyed atoms. The depth of the void was ~ 17 nm as seen from Fig. 1e-2. At an increased annealing temperature to 600 °C, the voids started to grow larger as shown in Fig. 1f–f-2. This can be correlated to the coalescence of the nearby voids due to the surface capillary forces. At the same time, the diffusing atoms were accumulated around the void edges, making large bumps and trough on the surface as depicted in the line profiles in Fig. 1f-2. Along with the increased temperature at 700 °C, the wiggly nanostructures were evolved as the void rims can be broken due to the Rayleigh-like stability as clearly evidenced by the AFM image in Fig. 1g and SEM image in Fig. 2a. As a result, the surface coverage was drastically decreased, and the height of nanostructure was significantly increased as compared with the preceding sample. Finally, at high annealing temperature between 800 and 900 °C, the isolated PdAuAg alloy NPs were formed along with the enhanced diffusion of alloy atoms as shown in Fig. 1h, i and Fig. 2b, c. The wiggly nanostructures were gradually becoming isolated and regular in shape due to the minimization of surface/interface energy as discussed [25]. Furthermore, the surface morphology of various nanostructures was analyzed by the RMS roughness (Rq) and surface area ratio (SAR) as shown in Fig. 2d and summarized in Additional file 1: Table S1. The Rq indicates the average height evolution of nanostructures, $\mathrm{Rq}=\sqrt{\frac{1}{\mathrm{n}}\sum \limits_1^{\mathrm{n}}{\mathrm{Z}}_{\mathrm{n}}^2}$, where the Zn is the height profile at each pixel. On the other hand, the SAR gives the percentile increment of surface area as $\mathrm{SAR}=\frac{A_g-{A}_s}{A_g}\times 100\%$, where the *A*_*g*_ is geometric (2D) and *A*_*s*_ is nanostructures surface area (3D), respectively. The Rq and SAR of corresponding nanostructures were gradually increased along with the annealing temperature, indicating the increased average height and surface area. In specific, the Rq was increased from 5.7 to 25.3 nm and the SAR was increased from 1.13 to 11.36% when the annealing temperature was varied between 400 and 900 °C. In addition, the EDS spectral analysis was implemented to analyze the elemental composition (Pd, Au, and Ag) of PdAuAg alloy nanostructures at various temperature as shown in Fig. 2e, f and Additional file 1: Figure S5. The EDS spectra suggest the presence of Pd, Au, and Ag with variant count as shown in Fig. 2e-1. Generally, the EDS counts signify the number of X-rays coming from the different orbits of atoms. The Au Mα1 peak counts are comparatively higher than that of Ag Lα1 and Pd Lα1 peak, which can be likely due to the higher atomic number of Au as compared to Ag and Pd. In addition, the EDS count plot in Fig. 2f displays that the Pd count was almost identical for all the samples annealed at different temperature. However, the Au count was similar up to 800 °C but slightly decreased at 900 °C, and the Ag count was similar up to 500 °C but gradually decreased between 600 and 900 °C. The reduction of Au and Ag counts can be due to the sublimation at specific vaporization temperatures [26–28]. Based on the vapor curve of Pd, Au, and Ag atoms, the Ag and Au atoms can be vaporized ~ 500 °C and ~ 800 °C, respectively, whereas the Pd cannot desorb within this annealing range, i.e., up to 900 °C [26–28]. The sublimation of Au and Ag can also affect the evolution of alloy NPs at high temperature such as configuration transition and volume reduction.

**Fig. 3:**
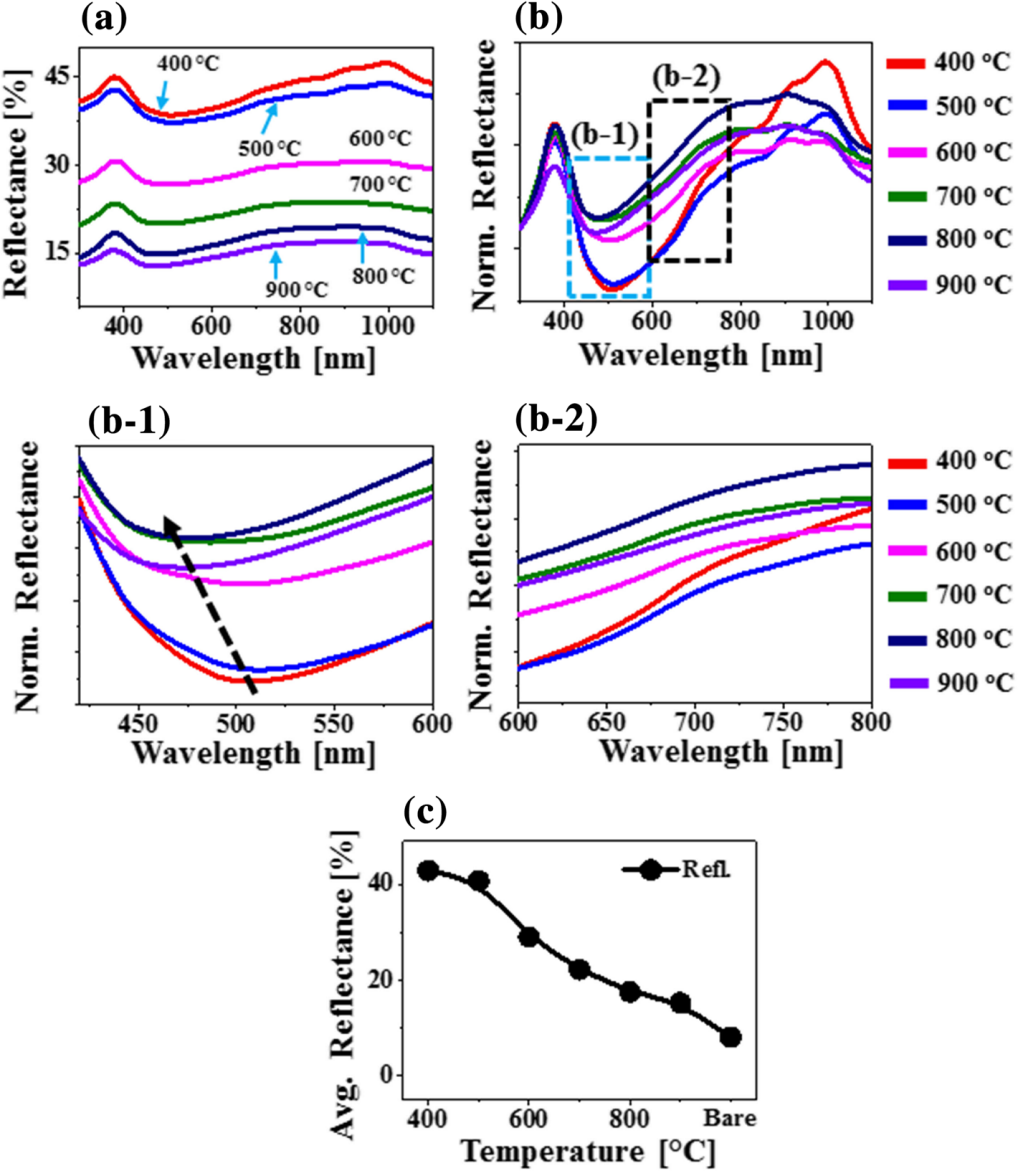
Reflectance spectra of corresponging PdAuAg nanostructures annealed at various temperature between 400 and 900 °C tri-layers. **a** Reflectance spectra. **b** Plot of average reflectance as a funtion of annealing temperature. **c** Normalized reflectance spectra at 300 nm. **c-1**–**c-2** Magnified reflectance spectra at specific locations to show the shift of dips and shoulders: boxed areas in **b**

Figure 3 shows the reflectance spectra of various PdAuAg alloy nanostructures on sapphire (0001) fabricated at different temperatures between 400 and 900 °C with 15-nm-thick Pd/Au/Ag tri-layers. In specific, the original (experimentally measured) reflectance spectra of all the alloy nanostructures are presented in Fig. 3a, which generally exhibited a peak in the UV region at ~ 380 nm, a dip in the visible region at ~ 500 nm, and a wide shoulder in the NIR region from ~ 900 to 1000 nm. In terms of the average reflectance, it was gradually reduced along with the evolution of isolated alloy NPs from the metallic tri-layers by annealing at elevated temperature as summarized in Fig. 3b. This can be correlated to the reduced surface coverage of the highly reflective metallic surface. Furthermore, the original spectra were normalized at 300 nm in order to analyze the spectral response more clearly such as trend of peaks and dips evolution in specific wavelength as shown in Fig. 3c. The normalized reflectance spectra clearly demonstrate the formation of strong peaks in the UV and NIR region and dips in the visible region, which can be correlated to the localized surface plasmon resonance (LSPR) of the alloy nanostructures. The reflectance peak in the UV region (~ 380 nm) can be attributed to the quadrupolar resonance and another peak in the NIR region is attributed to the dipolar resonance of the PdAuAg nanostructures [29, 30]. On the other hand, the reflectance in the visible region was sharply attenuated making a dip centered between 450 and 550 nm, which can be due to the absorption of the visible wavelength by the LSPR effect of alloy nanostructures. The UV peak showed quite consistent behavior whereas the visible dip and NIR shoulder were significantly modulated depending upon the surface morphology of PdAuAg nanostructures at various temperature, which were further explored by enlarging the corresponding sections as shown in Fig. 3c-1,–c-2. The absorption dip was gradually blue-shifted from ~ 510 to ~ 475 nm with the increased annealing temperature, which can be due to the decreased average size and increased spacing between alloy nanostructures [31]. Similarly, the reflectance shoulder between 600 and 800 nm also showed a distinct blue shift at increased temperature as shown in Fig. 3c-2. At the same time, the NIR shoulder became wider with the increased temperature, which can be correlated to the loss of Ag atoms due to the sublimation.

**Fig. 4:**
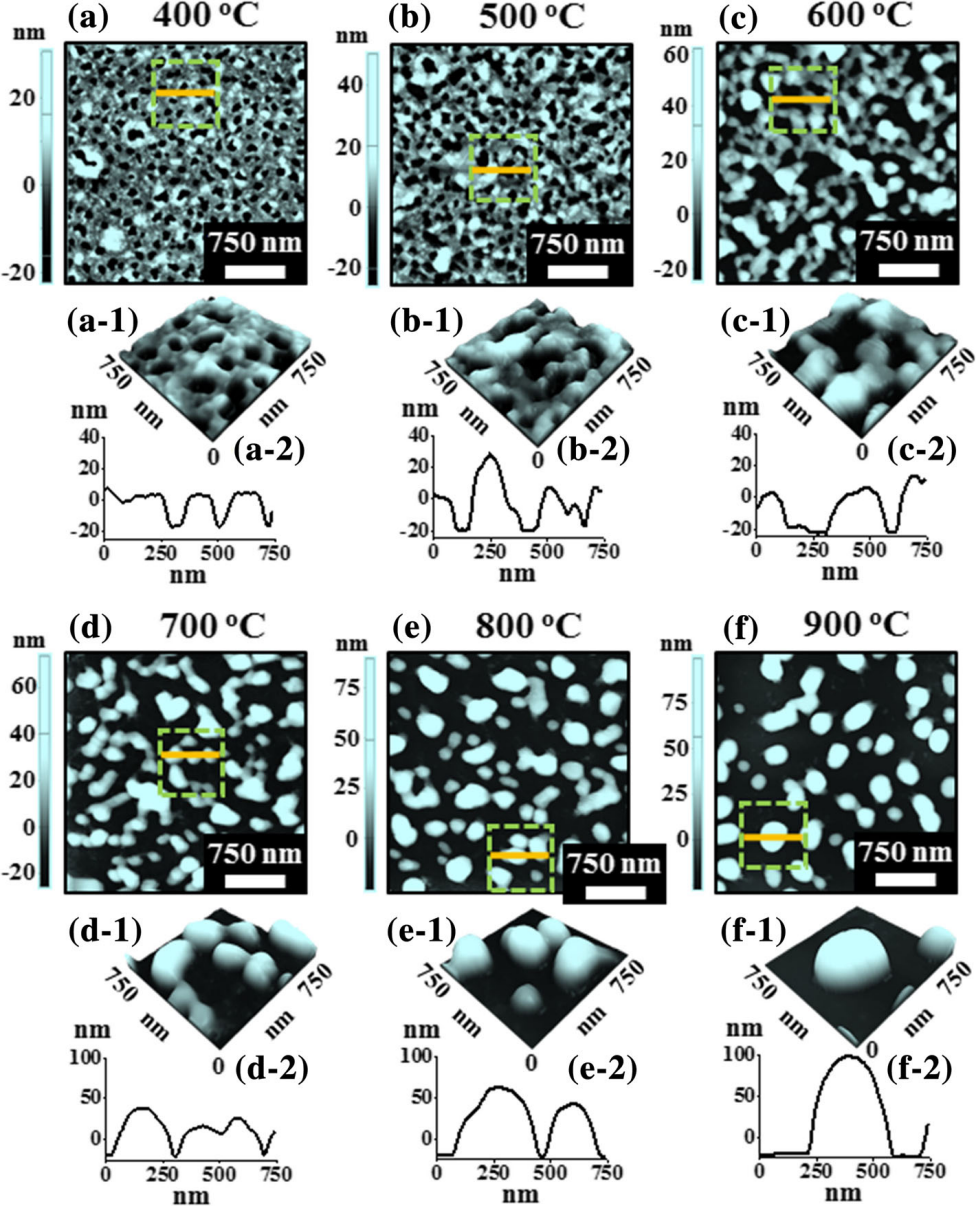
PdAuAg nanostructures based on the multi-layers of 15 nm Pd/Au/Ag films (15 layers) with 1 nm each. The annealing temperature was systematically varied between 400 and 900 °C for 450 s (multi-layers). **a**–**f** AFM top views (3 × 3 μm^2^). **a-1**–**f-1** Magnified AFM side views (750 × 750 nm^2^). **a-2**–**f-2** Cross-sectional line profiles

**Fig. 5:**
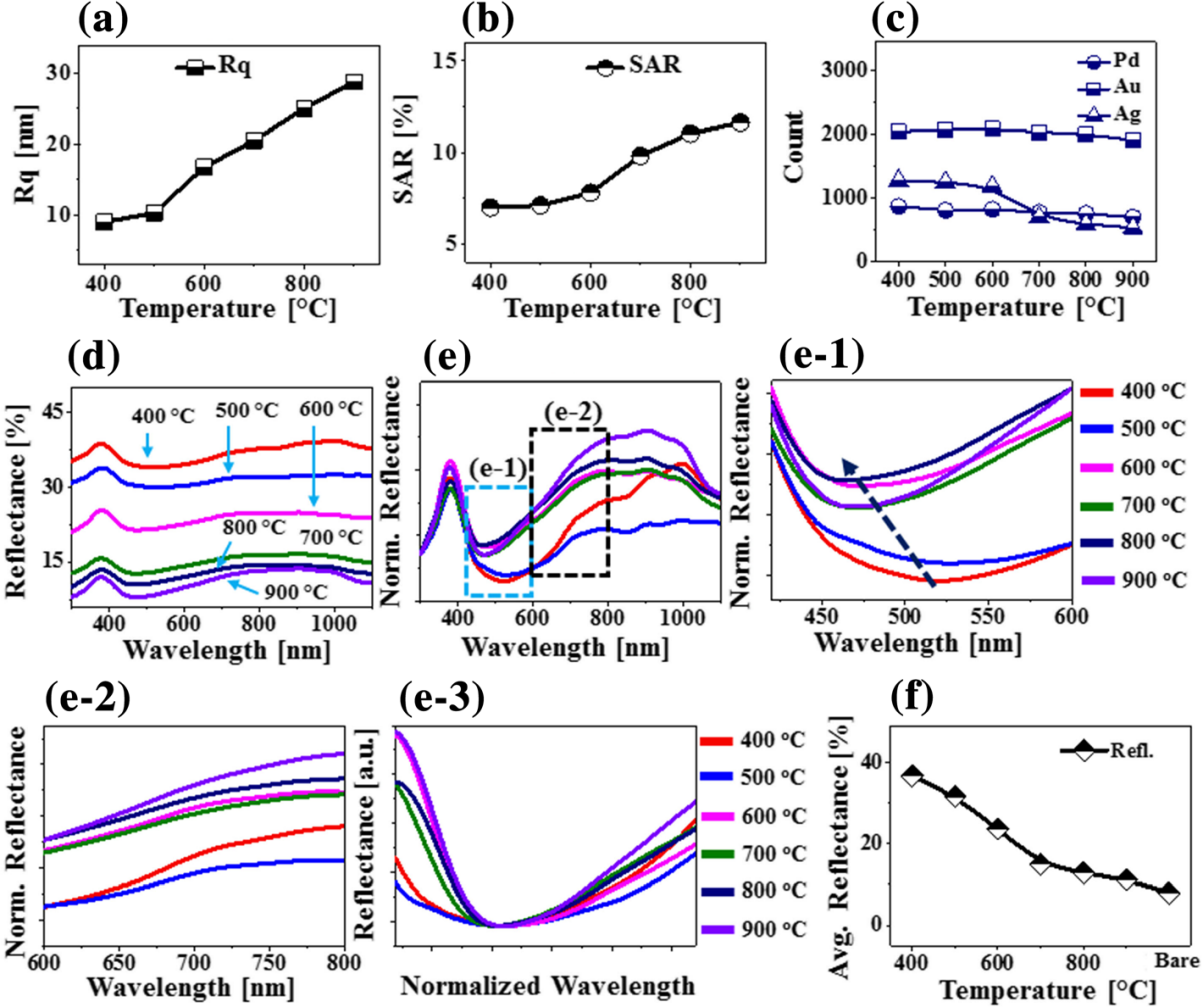
Morphological, elemental and optical analysis of corresponging PdAuAg nanostructures (multi-layers). **a**, **b** Plots of Rq and SAR. **c** Plots of Pd, Au, and Ag EDS counts. **d** Reflectance spectra. **e** Summary plot of average reflectance. **f** Normalized reflectance spectra at 300 nm. **f-1**–**f-2** Magnified regions boxed in **f**. **f-3** Normalized reflectance spectra to show the absorption dip width variation

Figure 4 shows the growth of PdAuAg alloy nanostructures with multi-layers of Pd, Au, and Ag films with the total thickness of 15 nm annealed under an identical growth condition as the previous set. Using the multi-layers film composition as shown in Fig. 1b, a sharp distinction of dewetting sequence was observed as compared with the tri-layer samples. More specifically, the dewetting process was significantly enhanced at low annealing temperature and resulted in the formation of more isolated and regular alloy NPs at high temperature. The individual thickness of constituent metallic films can significantly affect the dewetting process such that the inter-diffusion of atoms and alloying can be facilitated by the deposition of thinner layer. Therefore, by adapting the thinner and multi-layer composition of metallic thin films enhanced atomic diffusion, and hence higher dewetting rate can be expected [21]. In this case, the total film thickness was 15 nm that consisted of 15 layers of 1-nm-thick Pd, Au, and Ag by repeating the deposition as shown in Fig. 1b. As the overall diffusivity of the atoms can be enhanced along with the temperature, the dewetting process can be accelerated even at lower temperature. For instance, as shown in Fig. 4a, the large voids were formed even at 400 °C whose depth was ~ 15 nm and width was ~ 100 nm as clearly depicted by the line profile in Fig. 4a-2. This result clearly showed a sharp distinction from the tri-layer sample that only formed overgrown NPs at same temperature. Furthermore, the voids were gradually grown larger along with the increased annealing temperature between 500 and 600 °C due to the coalescence of adjacent voids as shown in Fig. 4b–b-2, c–c-2. During the temperature increment, the size of the voids was significantly increased owing to the enhanced diffusion and accumulation of alloy atoms as discussed. Furthermore, annealing at high temperature between 700 and 900 °C resulted more isolation between PdAuAg NPs as compared to the tri-layer samples. In particular, the connected nanostructures were formed at 700 °C as shown in Fig. 4d–d-2, which were more fragmented and slightly taller than tri-layer sample. Consequently, the irregular configuration was gradually transformed into the spherical NPs along with the temperature as shown in Fig. 4e, f. This shape transformation of the isolated NPs can be governed by the surface energy minimization and equilibrium configuration [32]. On the other hand, due to the coalescence of adjacent NPs, the NPs’ density was gradually reduced whereas size was increased. In addition, the Rq, SAR, elemental, and optical analysis of corresponding PdAuAg nanostructures fabricated with multi-layer samples are presented in Fig. 5a–c. The Rq and SAR were gradually increased with the annealing temperature as shown in Fig. 5a, b. As compared to the tri-layers sample, the Rq and SAR values were generally higher for multi-layers sample because of the formation of larger voids and alloy nanostructures. Furthermore, the plot of Pd, Au, and Ag count in Fig. 5c demonstrated similar trend of counts with the tri-layers samples because of the total deposited amount of each metal was identical. The reflectance spectra of PdAuAg alloy nanostructures in this set are presented in Fig. 5d–f. The overall trend of reflectance spectra was quite similar with the previous set as shown in the original and normalized reflectance spectra in Fig. 5d, f, respectively. However, the specific peak intensity, position and shift behavior were significantly altered. This set of samples also exhibited a peak at ~ 380 nm, a dynamic dip in the visible region and a wide shoulder in the NIR region due to the LSPR effect of the PdAuAg alloy NPs as discussed. Similarly, the average reflectance was gradually decreased by the formation of isolated alloy NPs from the metallic multi-layers as shown in Fig. 5e. Generally, the average reflectance was slightly lower than the previous set, which can be associated with the much reduced surface area by the enhanced dewetting with the multi-layer scheme. The normalized and enlarged reflectance spectra clearly demonstrate the dynamic spectral shape, peak/dip position and width as shown in Fig. 5f-1–f-3. Along with the gradual evolution of voids, connected nanoclusters and isolated alloy NPs between 400 and 900 °C, the absorption dip was gradually blue-shifted from ~ 520 to ~ 465 nm [31]. This blue shift of absorption dip can be due to the gradual reduction of average size as the connected nanoclusters were developed into isolated spherical NPs as discussed. Furthermore, the reflectance shoulders were generally blue shifted along with the increased annealing temperature. The absorption dip observed in the visible region became narrower between 400 and 900 °C along with the improved size uniformity of alloy nanostructures [31, 33].

**Fig. 6:**
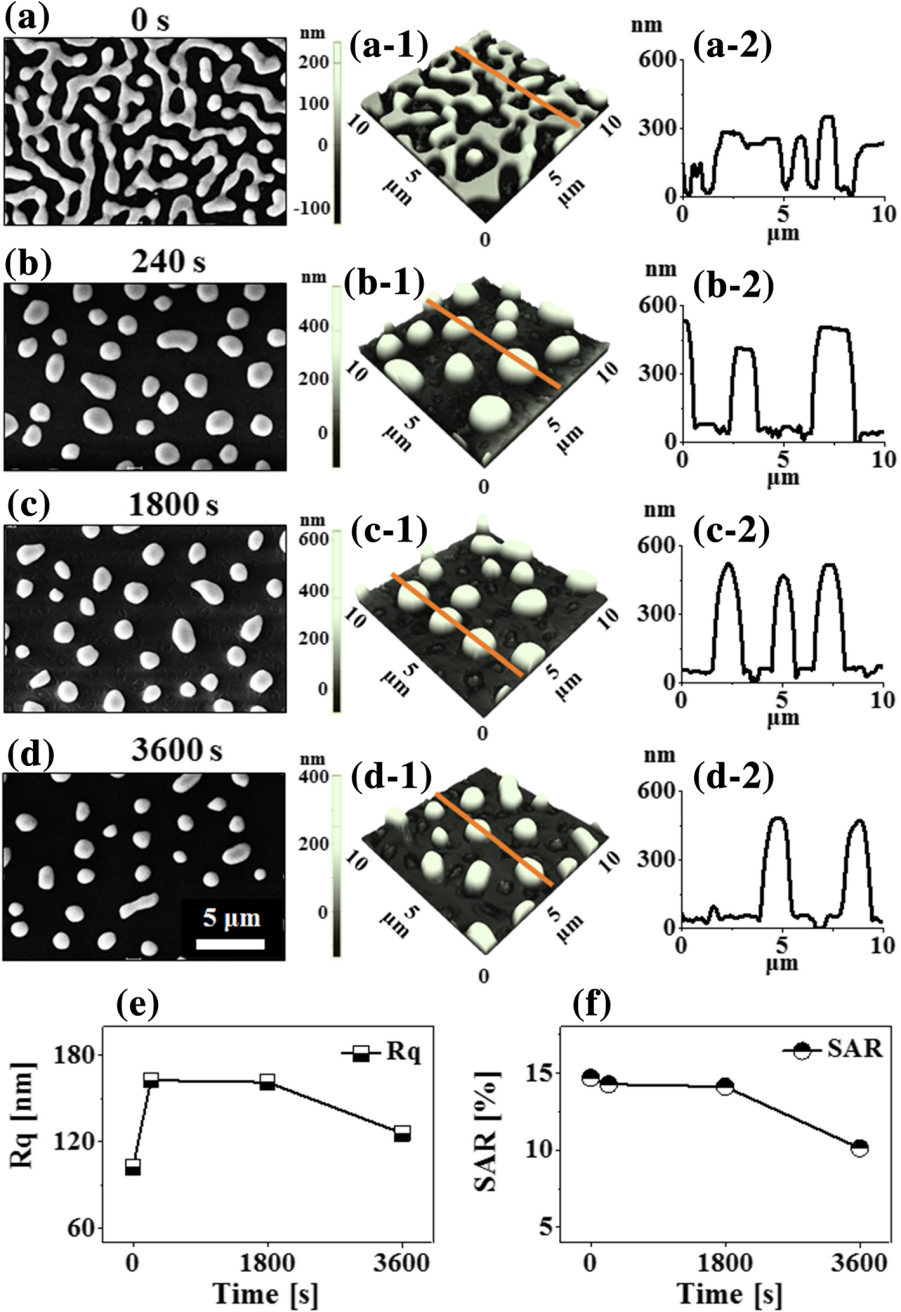
Fabrication of bi-metallic PdAg nanostructures on saaphire (0001) by the control of annealing time with the Pd_150 nm_/Ag_80 nm_ bi-layers annealed at 850 °C (bi-layers). **a**–**d** SEM images. **a-1**–**d-1** Corresponding AFM side-views (10 × 10 μm^2^). **a-2**–**d-2** Cross-sectional line profiles. **e**, **f** Plots of Rq and SAR

Figure 6 shows the evolution of PdAg alloy nanostructures on sapphire (0001) by the control of annealing time at a fixed annealing temperature of 850 °C with the Pd_150 nm_/Ag_80 nm_ bi-layer. This set of samples was prepared to observe the time evolution of bimetallic alloy NPs by using the highest (Ag) and lowest diffusivity (Pd) metals among Au, Ag, and Pd. As shown by the SEM and AFM images in Fig. 6, the wiggly connected nanostructures were gradually transformed into the isolated round NPs along with the increased annealing time at fixed temperature. Furthermore, this result showed a sharp distinction on the morphological evolution of Pd NPs from our previous work at similar growth conditions and thickness of Pd film [34]. In which, the 150-nm-thick Pd film only showed the formation of voids at 850 °C, which can be due to the low diffusivity and dewetting rate of thick Pd film [34]. This clearly indicates that the dewetting rate of the low diffusivity metals can be enhanced by the addition of high diffusivity metal films [21, 35]. The dewetting enhancement can be correlated to the interdiffusion and alloying of Pd and Ag atoms, which increases the overall diffusivity of atoms [36]. As shown in Fig. 6a, the wiggly nanostructures were formed at 850 °C for 0 s as the significant dewetting was already occurred. The typical height of the nanostructures was ~ 300 nm as clearly demonstrated by the cross-sectional line-profile in Fig. 6a-2. Along with the increased annealing time to 240 s at fixed temperature, the connected nanostructures were transformed into the isolated NPs of ~ 500 nm average height. This morphological transformation can be correlated to the Rayleigh-like instability and surface energy minimization along with the enhanced diffusion of alloy atoms. Furthermore, the gradual size decrement of isolated alloy NPs was observed when the annealing duration of further increased between 1800 and 3600 s, which can be due to the sublimation of Ag atoms as discussed before [37]. The analysis of Rq and SAR was performed on large-scale AFM images as shown in Fig. 6e, f and was summarized in Additional file 1: Table S4. The Rq was sharply increased from 0 to 240 s by the formation of isolated NPs from large connected nanostructures. However, it was gradually decreased between 1800 and 3600 s as the size of NPs was reduced due to the sublimation of Ag. Meanwhile, the SAR was gradually reduced with the increased annealing time due to the gradual decrement of the surface area of PdAg alloy NPs.

**Fig. 7:**
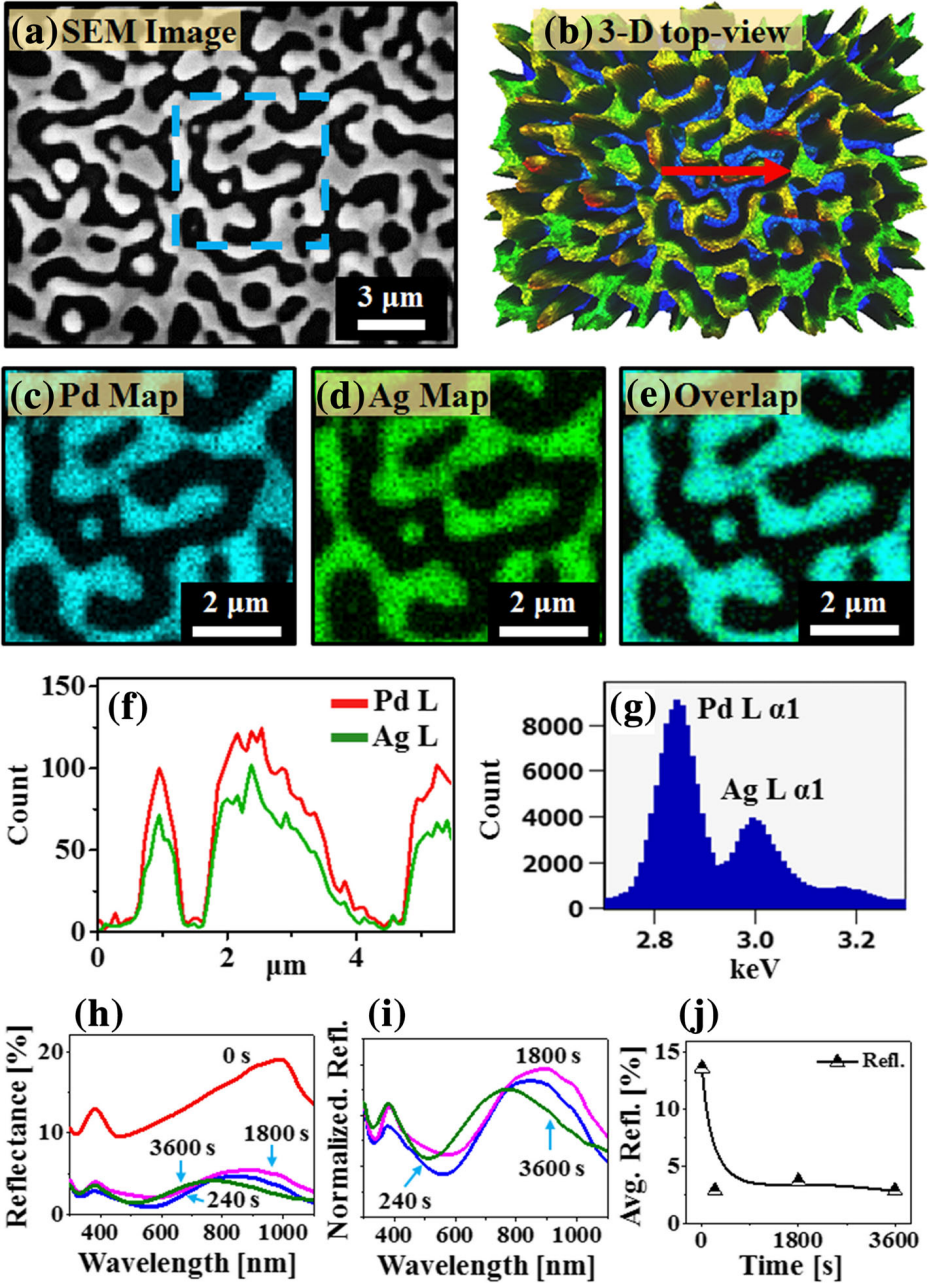
Elemental and optical analysis of PdAg bi-metallic nanostructures (bi-layers). **a** SEM image of the 0 s sample. **b** Top view of corresponding 3D map. **c**–**e** Maps of Pd, Ag and Pd and Ag together, boxed region in **a**. **f** EDS spectral line profiles of Pd and Ag, arrow location in **b**. **g** EDS spectrum of the 0 s sample. **h** Reflectance spectra of various PdAg nanostructures. **i** Normalized reflectance spectra at 300 nm. **j** Plot of average reflectance

Figure 7 shows the elemental and optical properties of corresponding PdAg nanostructures fabricated with Pd_150 nm_/Ag_80 nm_ bi-layer. The elemental analysis was performed on the wiggly connected PdAg nanostructures fabricated at 850 °C for 0 s as shown in Fig. 7a–g. The SEM image, 3D map, separate elemental maps, and EDS line profiles clearly demonstrated the elemental distribution of wiggly alloy nanostructures. For the detail analysis, the magnified Pd, Ag, and overlap map are demonstrated in Fig. 7c–e, which are acquired from the SEM image marked with blue rectangle. The Pd, Ag, and overlap maps were matched well, which indicates the formation of alloyed PdAg nanostructures. Furthermore, the elemental line-profile and EDS spectrum of PdAg nanostructure are shown in Fig. 7f–g. In the spectral line-profile, the Pd and Ag were originated from the PdAg nanostructure, which showed the comparatively higher intensity of Pd. This can be due to the high concentration of Pd atoms in the alloy NPs as the Ag were gradually sublimation. The corresponding reflectance spectra of PdAg nanostructures are presented in Fig. 7h–j. Depending upon the morphology of PdAg alloy nanostructures at various annealing time, the reflectance spectra showed a sharp distinction. By the formation of widely spaced large bimetallic PdAg nanostructures on sapphire, the absorption or reflectance can be significantly enhanced at specific wavelength as seen in the spectral shape of reflectance. For example, at 0 s, small peak at ~ 380 nm, strong absorption dip at ~ (500–600) nm, and another reflectance peak at NIR were developed due to the surface plasmon resonance of PdAg nanostructures as shown in Fig. 7h. Along with the formation of isolated PdAg alloy NPs between 240 and 3600 s, the NIR peak position and intensity were varied. In addition, the reflectance spectra of PdAg NPs between 240 and 3600 s were normalized at 300 nm as shown in Fig. 7i. The normalized spectra clearly revealed that the dipolar resonance peak was blue-shifted from ~ 990 to below 850 nm along with the reduction of NPs size from 240 to 3600 s as discussed [31]. In terms of average reflectance, the reflectance spectra at 0 s exhibited the highest reflectance and later significantly decreased with the formation of isolated NPs as shown in Fig. 7j. The average reflectance between 240 and 3600 s was almost similar and ~ 3%, which can be likely due to the highly absorbing nature of widely spaced PdAg NPs. Such behavior was also observed with monometallic Pd NPs, in which the wide spacing Pd NPs demonstrated the reduced reflectance in VIS region in the previous study [34].

## Conclusions

The fabrication of bi- and tri-metallic alloy nanostructures of Pd, Ag, and Au has been successfully demonstrated on sapphire (0001) via the solid-state dewetting. The various surface morphology of the alloy nanostructures were obtained by controlling the annealing temperature, time, and deposition scheme such as bi-, tri-, and multi-layers arrangement. The gradual evolution of over-layered NPs, voids, wiggly nanostructures, and isolated PdAuAg alloy NPs was observed by the annealing of 15-nm-thick Pd/Au/Ag tri-layers. In contrast, the multi-layers films of same thickness (15 nm) demonstrated significantly enhanced overall dewetting at identical annealing temperature such that the voids were evolved at lower temperature and well-spaced regular alloy NPs obtained at higher temperature, which was attributed to the enhanced inter-diffusion and alloying with thinner layers. Furthermore, depending upon the control of annealing time with the Pd_150 nm_/Ag_80 nm_ bi-layer, the configuration transition from the wiggly connected nanostructure geometry to the isolated PdAg alloy NPs was observed along with the enhanced diffusion of alloyed atoms. The overall growth of the alloy NPs was discussed based on the solid-state dewetting process in conjunction with surface diffusion, interdiffusion, alloy formation, Rayleigh-like instability, and energy minimization. The optical properties of such alloy NPs were investigated by the reflectance spectra, which revealed the formation of absorption dip, quadrupolar, and dipolar resonance peaks at specific wavelength based on the dynamic LSPR effect of different alloy NPs. Both the bi- and tri-metallic alloy NPs exhibited the strong absorption in the visible region and dipolar and quadrupolar resonance peaks in the NIR and UV region, respectively. The quadrupolar was seems to be insensitive with the morphological variation whereas the absorption dip and dipolar peaks were gradually blue shifted with the formation of isolated and smaller alloy NPs.

## Additional file

Additional file 1: **Figures S1–S13.** Supplementary materials include additional AFM images, SEM images, EDS spectra, Raman spectra of various PdAuAg and PdAg alloy nanostructures. **Tables S1–S4.** Summary of Rq, Ra, SAR, average reflectance and intensity, peak position of Raman band A1_g_ of various PdAuAg, and PdAg alloy nanostructures. (DOCX 26443 kb)

## Abbreviations

AFM: Atomic force microscope
EDS: Energy-dispersive X-ray spectroscope
LSPR: Localized surface plasmon resonance
NIR: Near infrared
NPs: Nanoparticles
PLD: Pulsed laser deposition
Rq: RMS roughness
SAR: Surface area ratio
SEM: Scanning electron microscope
SSD: Solid-state dewetting
UV: Ultra-violet
VIS: Visible
