# Supplementary material for: Modulation of Morphology and Optical Property of Multi-Metallic PdAuAg and PdAg Alloy Nanostructures
Source: Nanoscale Res Lett. 2018 May 16;13:151. doi: 10.1186/s11671-018-2551-0 (PMC5955875; doi:10.1186/s11671-018-2551-0)
Supplement: Supplementary file 1 — Figures S1–S13. Supplementary materials include additional AFM images, SEM images, EDS spectra, Raman spectra of various PdAuAg and PdAg alloy nanostructures. Tables S1–S4. Summary of Rq, Ra, SAR, average reflectance and intensity, peak position of Raman band A1g of various PdAuAg, and PdAg alloy nanostructures. (DOCX 26443 kb) [file 11671_2018_2551_MOESM1_ESM.docx]

**Supporting Information to**

***Modulation of Morphology and optical property of multi-metallic PdAuAg and PdAg alloy nanostructures***

**Puran Pandey^1^, Sundar Kunwar^1^, Mao Sui^1^, Sushil Bastola^1^, and Jihoon Lee^1,2^***

^1^ College of Electronics and Information, Kwangwoon University, Nowon-gu Seoul 01897, South Korea

^2^ Institute of Nanoscale Science and Engineering, University


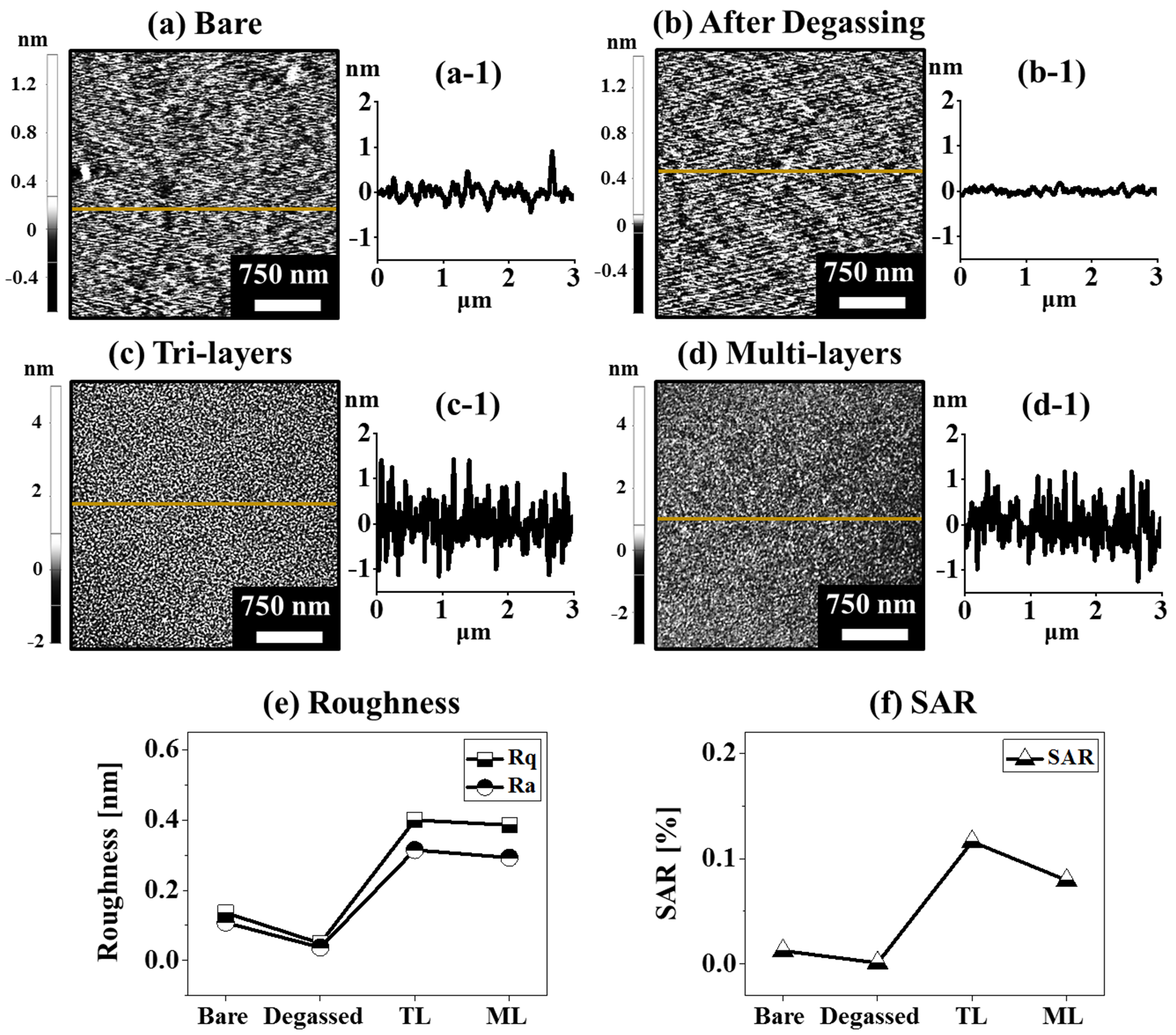


**Fig. S1** Surface morphologies of bare sapphire (0001) and deposited samples. (a) Bare sapphire. (b) Sapphire after degassing at 600 ^o^C for 30 minutes. (c) PdAuAg deposition, 15 nm of Pd/Au/Ag with 5 nm each layer (Tri-layers). (d) PdAuAg deposition, 15 nm of multilayer Pd/Au/Ag (15 layers) with 1 nm each (Multi-layers). (a) – (d) AFM top-views (3 × 3 µm^2^). (a-1) – (d-1) Corresponding cross-sectional line-profiles. (e) – (f) Plots of corresponding Rq, Ra and SAR.


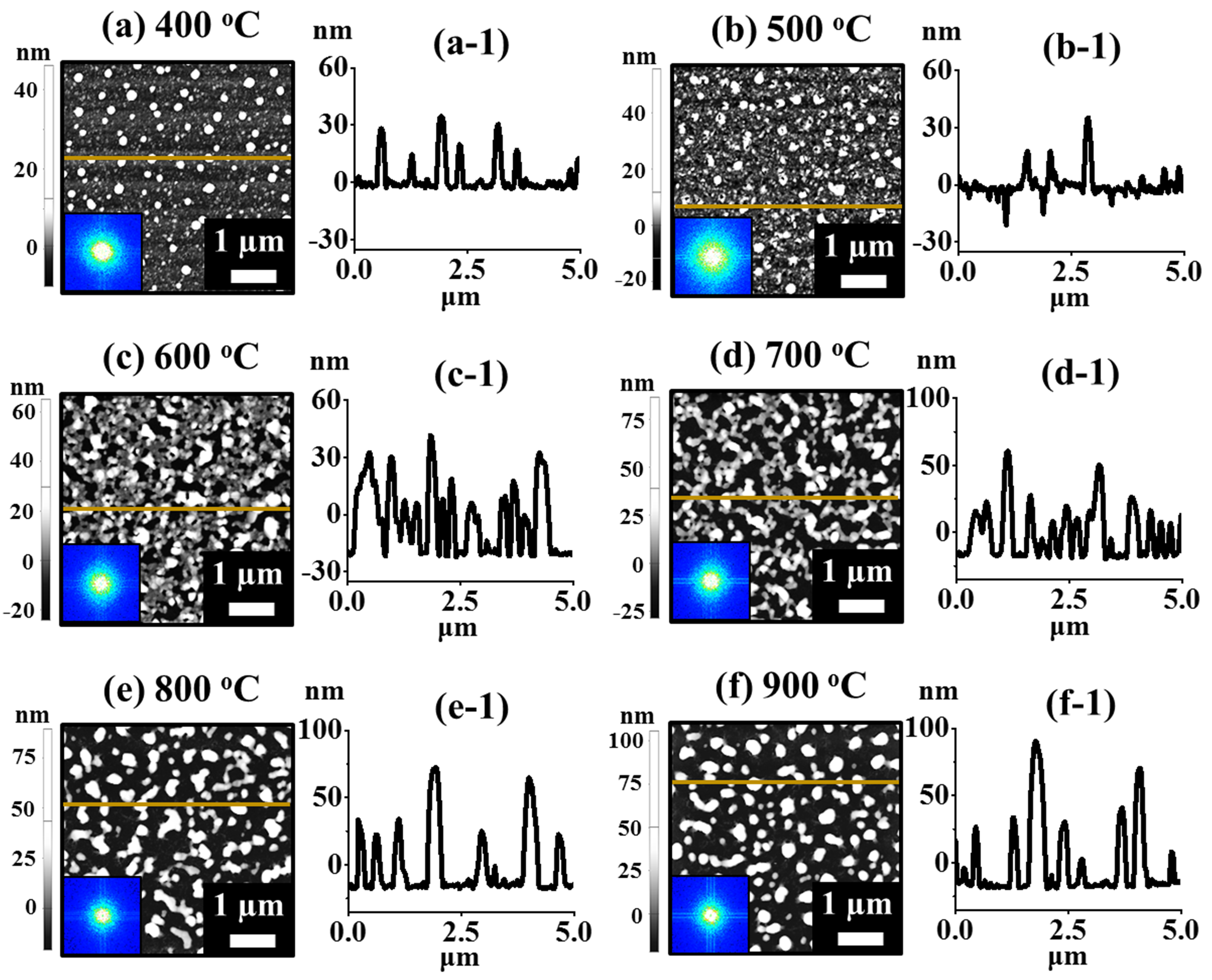


**Fig. S2** (a) – (f) Large scale AFM images (5 × 5 µm^2^) of PdAuAg trimetallic nanostructures annealed at various temperature with the 15 nm of Pd/Au/Ag with 5 nm each layer (Tri-layers). (a-1) – (f-1) Cross-sectional line-profiles. Insets in the AFM top-views are FFT power spectra.


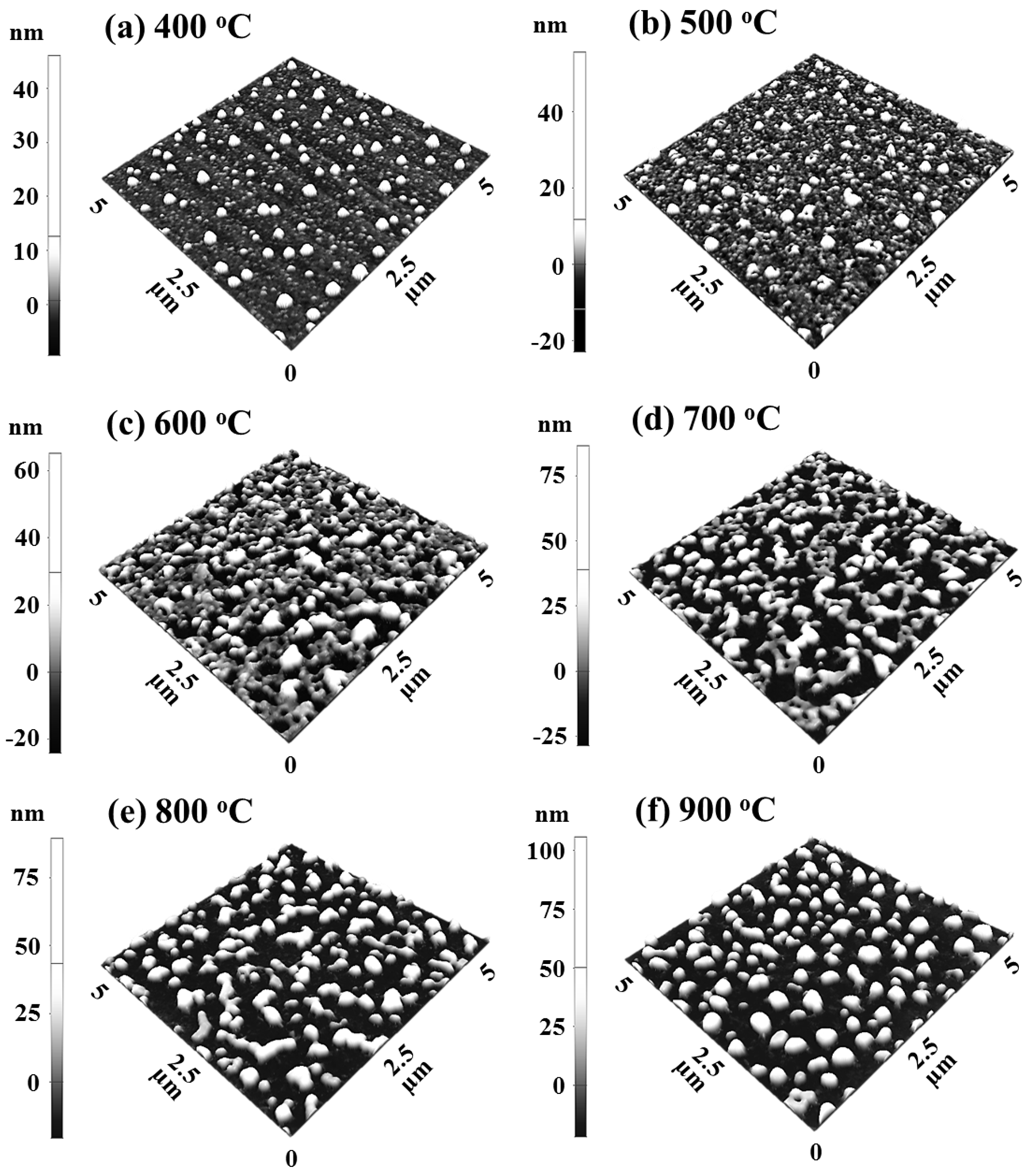


**Fig. S3** AFM side-views (5 × 5 µm^2^) of corresponding tri-metallic PdAuAg nanostructures based on the 15 nm of Pd/Au/Ag with 5 nm each (Tri-layers) annealed between 400 and 900 ^o^C.


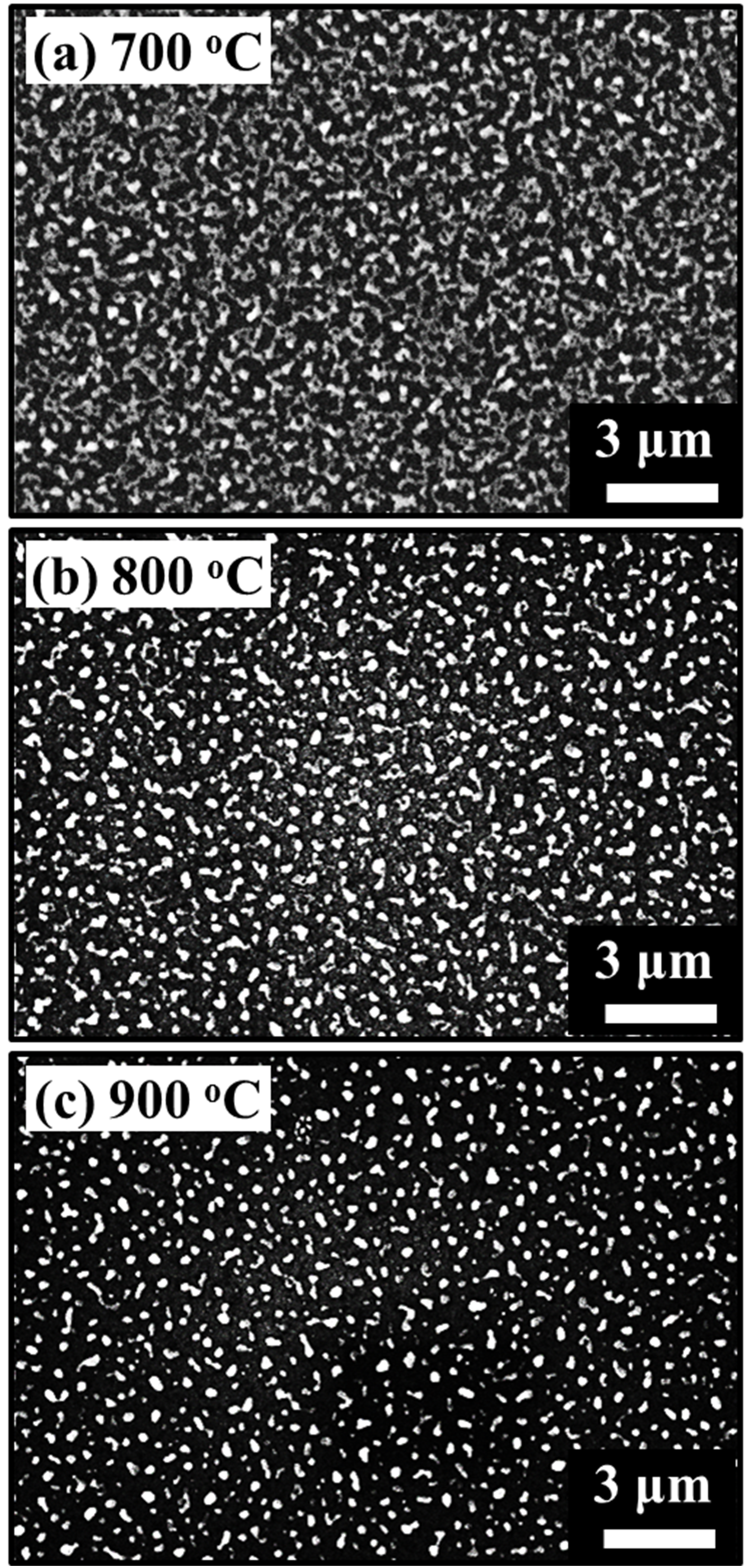


**Fig. S4** SEM images of PdAuAg nanostructures annealed at high temperature between 700 and 900 ^o^C (Tri-layers).


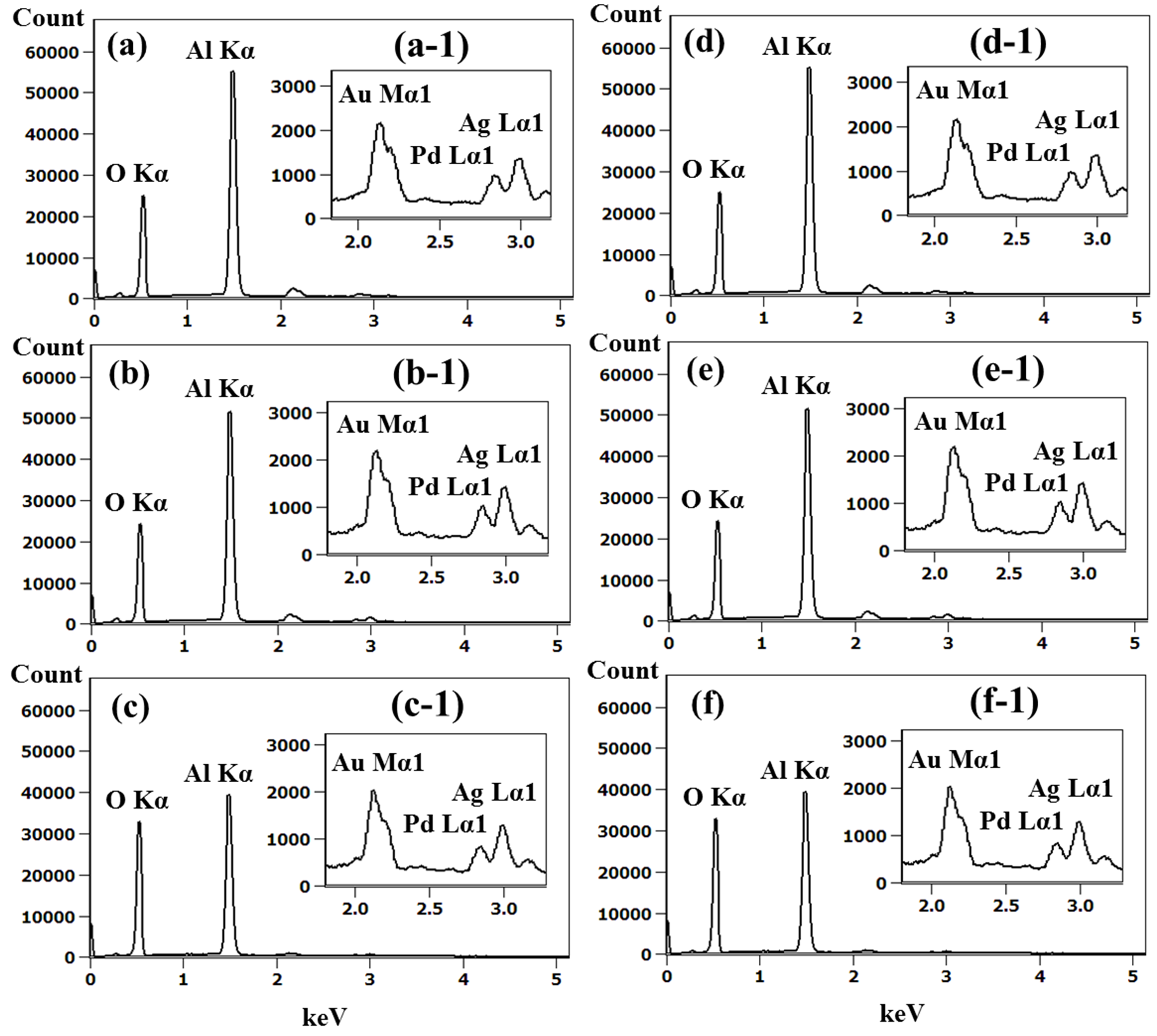


**Fig. S5** (a) – (f) EDS spectra of corresponding tri-metallic PdAuAg nanostructures annealed between 400 and 900 ^o^C. (a-1) – (f-1) Enlarged spectra to show the distinct peaks of Pd, Au and Ag (Tri-layers).


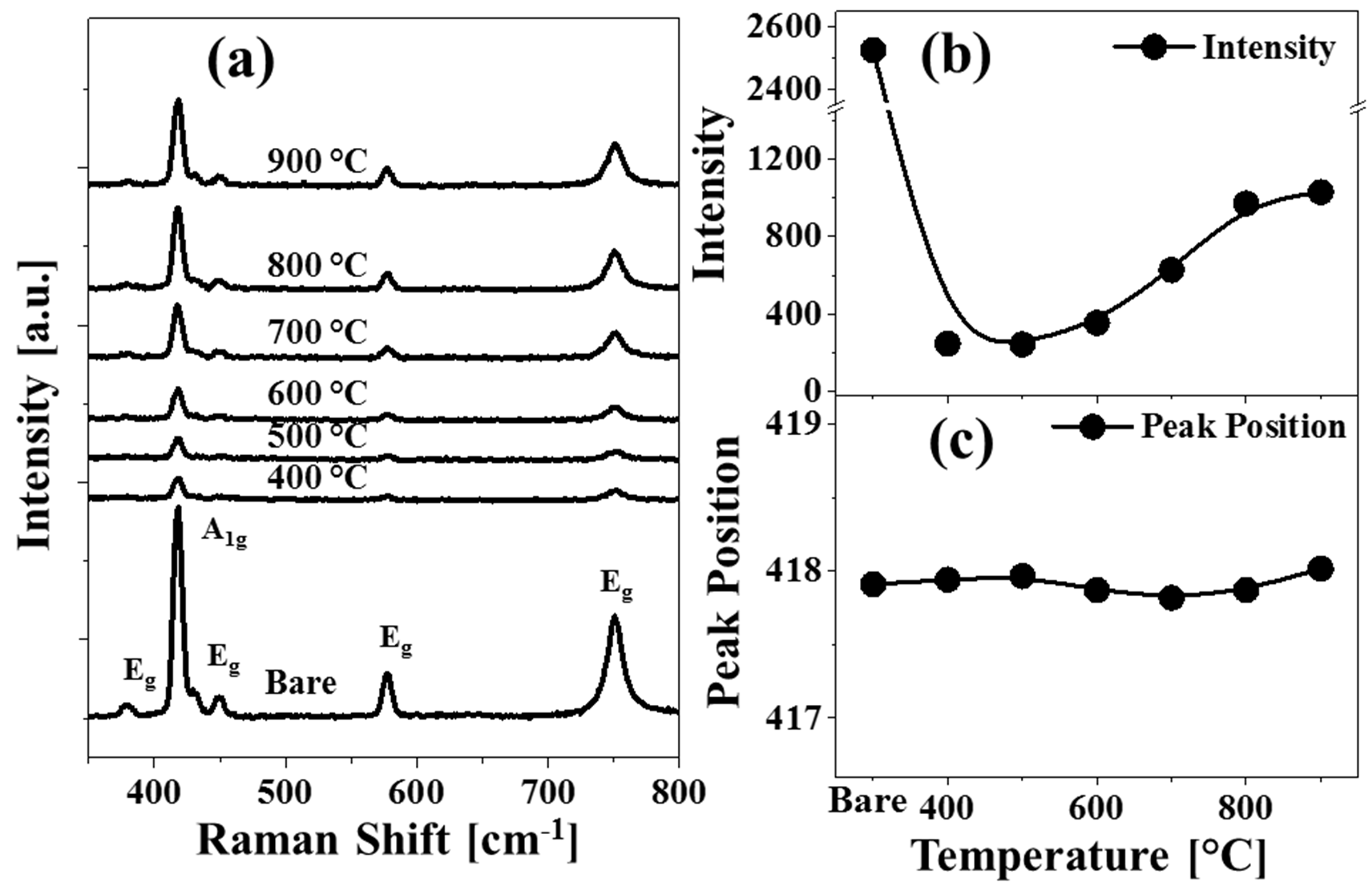


**Fig. S6** (a) Raman spectroscopy of various tri-metallic PdAuAg nanostructures (Tri-layers). (b) – (c) Plots of intensity and peak position of A_1g_ Raman peaks.


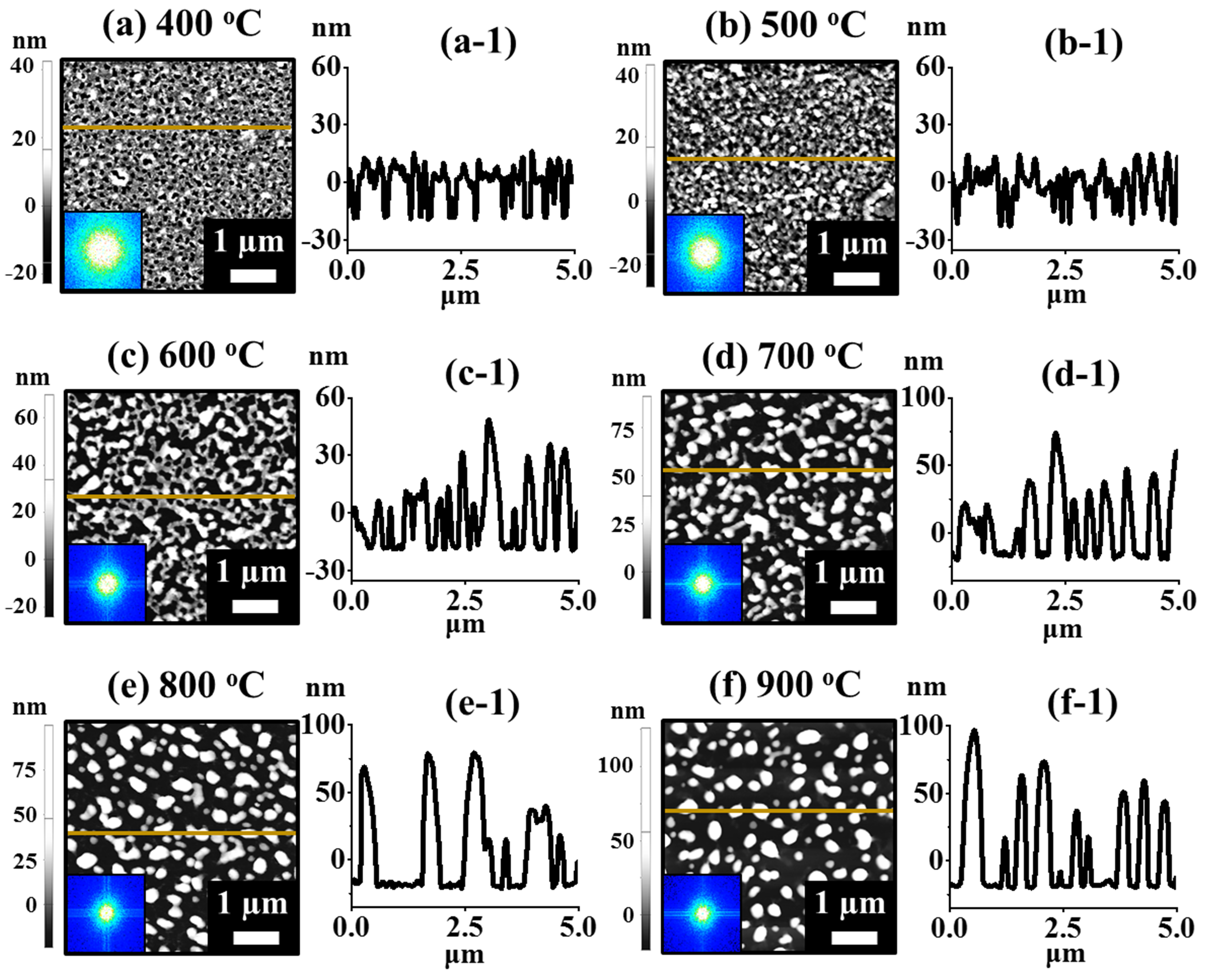


**Fig. S7** PdAuAg tri-metallic nanostructures with the tri-layer deposition annealed between 400 and 900 ^o^C; form void, connected nanostructures to well separated nanostructures (Multi-layers). (a) – (f) AFM top-views (5 × 5 µm^2^). (a-1) – (f-1) Cross-sectional line-profiles. (Insets) FFT power spectra.


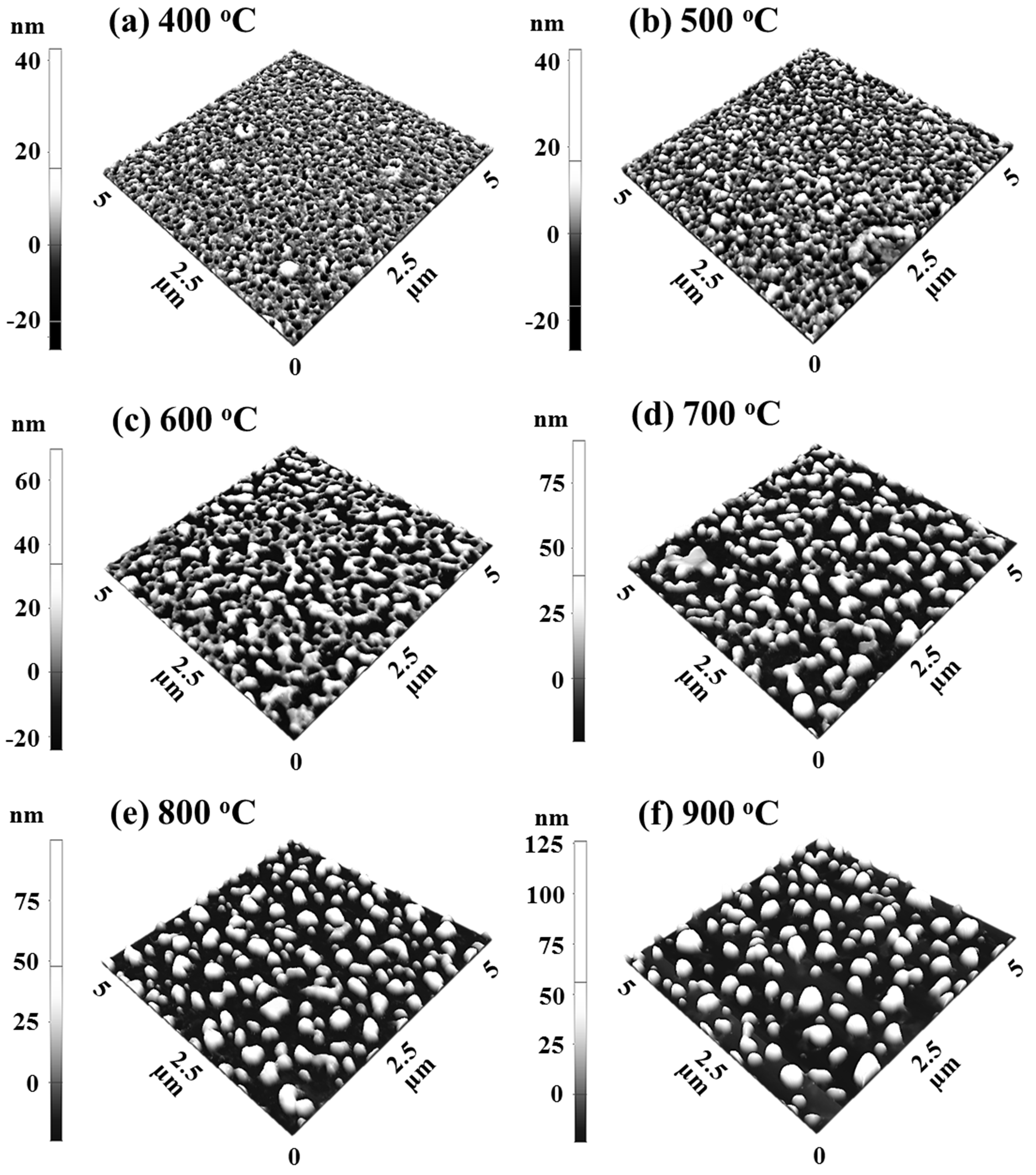


**Fig. S8** AFM side-views (5 × 5 µm^2^) of corresponding tri-metallic PdAuAg nanostructures annealed between 400 and 900 ^o^C (Multi-layers).


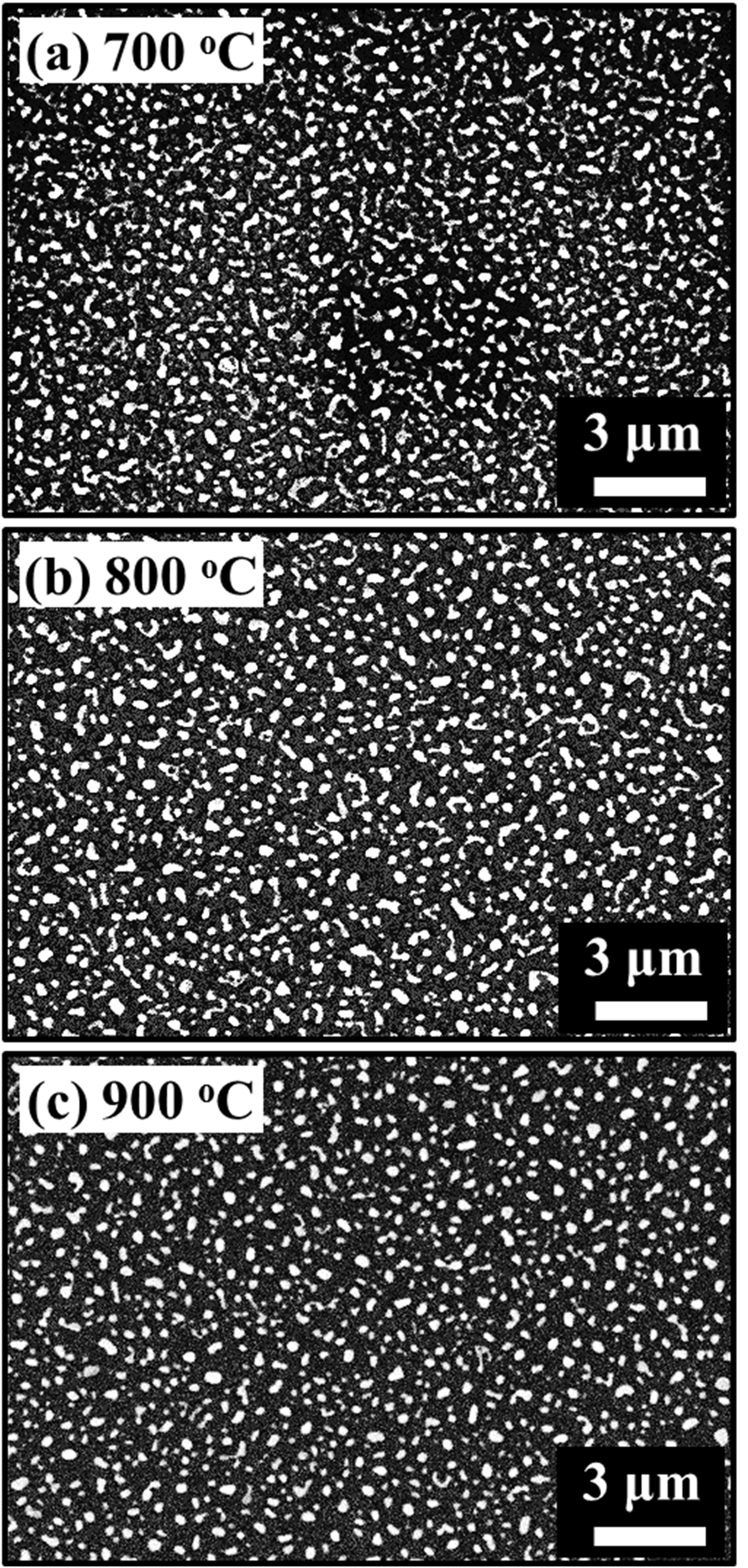


**Fig. S9** Large scaled SEM images of tri-metallic PdAuAg nanostructures annealed at high temperature between 700 and 900 ^o^C (Multi-layers).


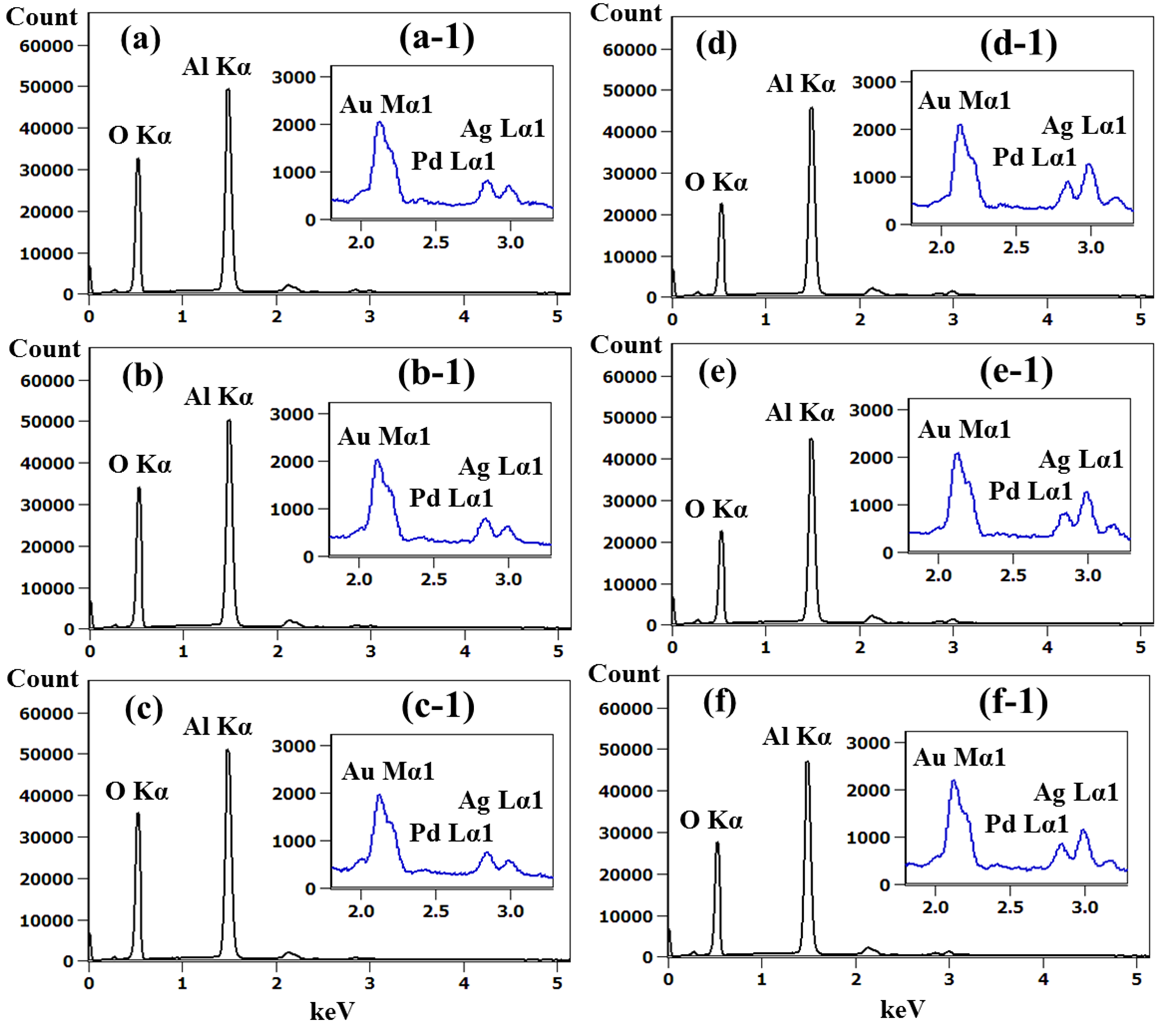


**Fig. S10** (a) – (f) EDS spectra of corresponding PdAuAg nanostructures annealed between 400 900 ^o^C (Multi-layers). (a-1) – (f-1) Enlarged EDS spectra.


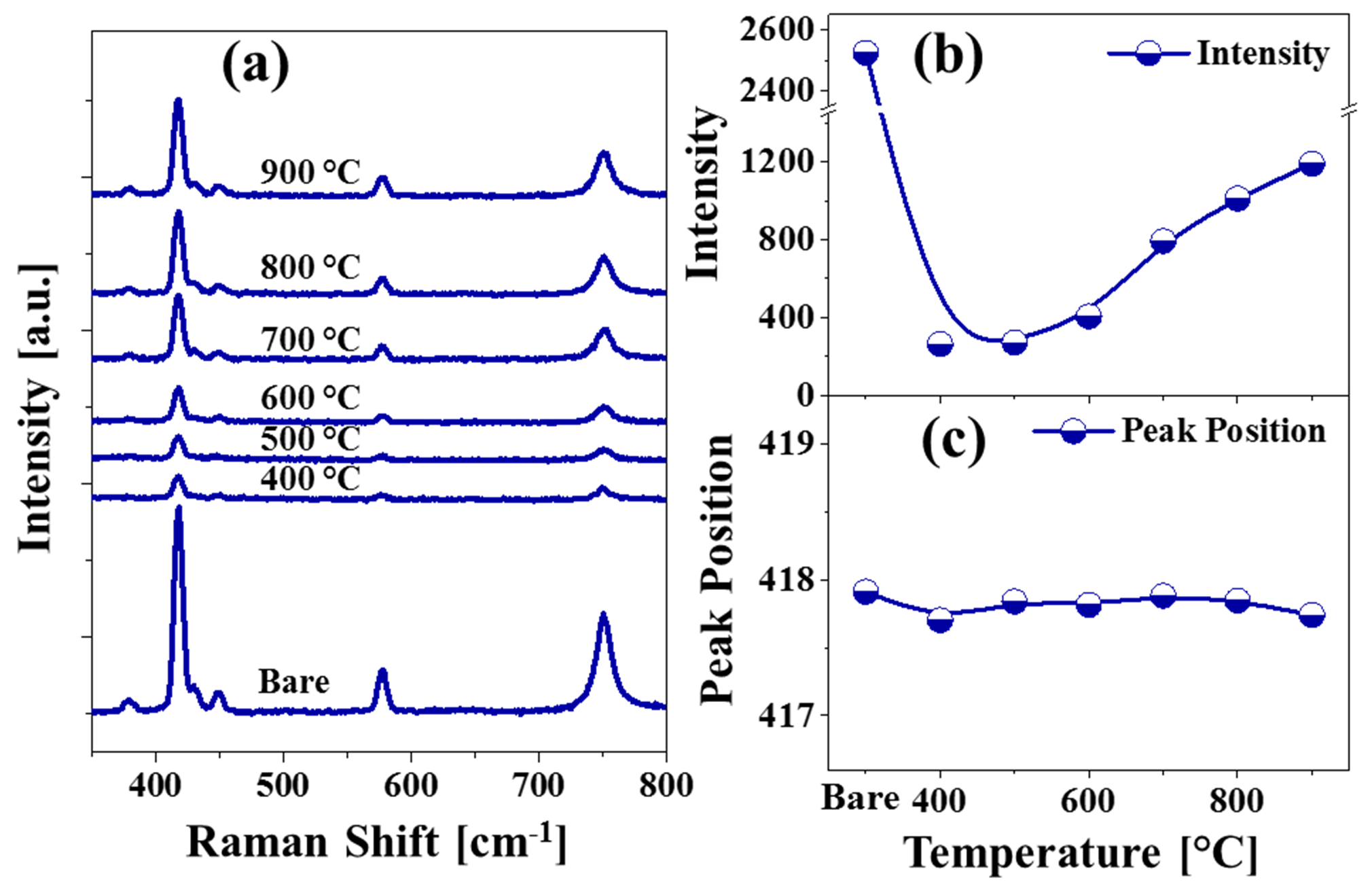


**Fig. S11** (a) Raman spectroscopy of tri-metallic PdAuAg nanostructures fabricated (Multi-layers). (b) – (c) Plots of intensity and peak position of A_1g_ Raman peaks.


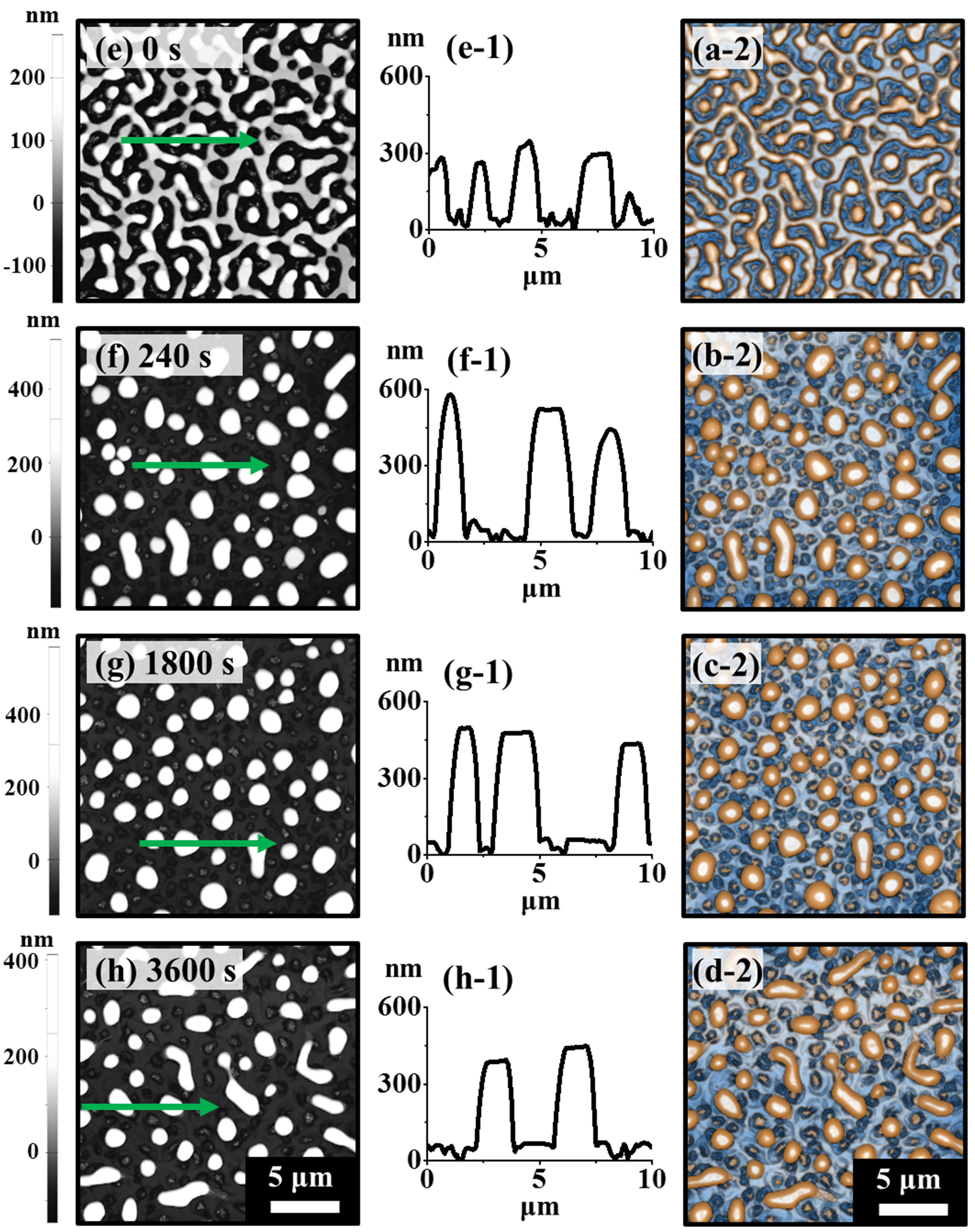


**Fig. S12** Evolution of bi-metallic PdAg nanostructures by the control of annealing time with the Pd_150 nm_ / Ag_80 nm_ bilayers at the annealing temperature of 850 ^o^C on sapphire (0001) (Bi-layers). (a) – (d) 2-D AFM top-views of 20 × 20 µm^2^. (a-1) – (d-1) Corresponding line-profiles. (a-2) – (d-2) Corresponding 3-D top-views showing the background clearer.


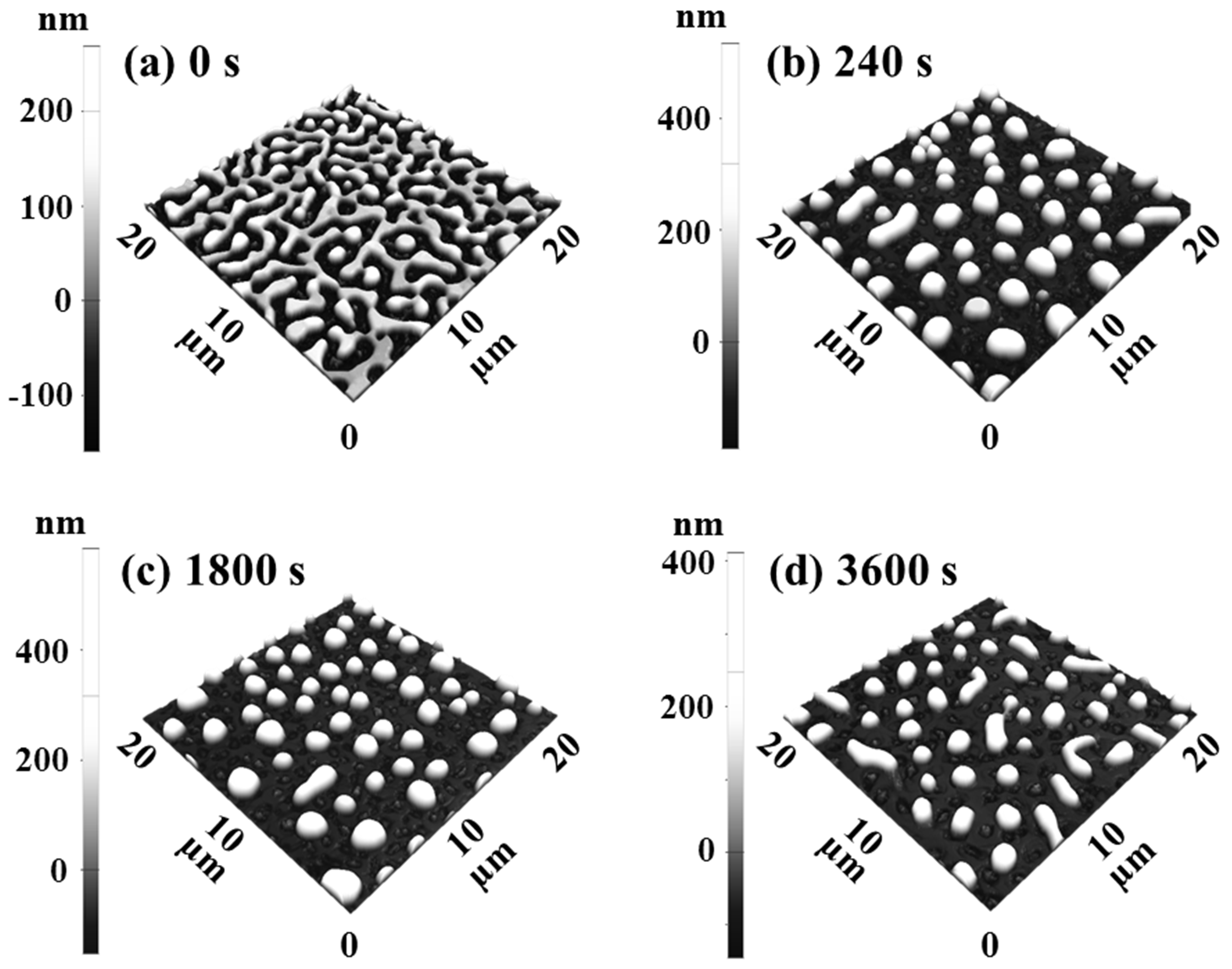


**Fig. S13** AFM side-views (20 × 20 µm^2^) of Pd-Ag nanostructures fabricated with the Pd_150 nm_ / Ag_80 nm_ bilayers at 850 ^o^C (Bi-layers).

**Table S1** Summary of Rq, Ra and SAR of various PdAuAg nanostructures by the contorl of anneling temperature between 700 and 900 ^o^C with the tri-layers and multilayers on sapphire (0001) . The Rq indicates the average height evolution of nanostructures, $Rq=\sqrt{\frac{1}{n}\sum_{1}^{n} Z_{n}^{2}}$ , where the Zn is the height profile at each pixel. On the other hand, the SAR gives the percentile increment of surface area as: $SAR= \frac{A_{g}-A_{s}}{A_{g}} \times100 \%$ , where the A_g_ is geometric (2D) and A_s_ is nanostructures surface area (3D) respectively

|  | **Tri-layers (15 nm)** | | **Multi-layers (15 nm)** | |  |
| --- | --- | --- | --- | --- | --- |
| **Temperature [^o^C]** | **Rq [nm]** | **SAR [%]** | **Rq [nm]** | **SAR [%]** | |
| **400** | 5.78 | 1.13 | 8.99 | 7.01 | |
| **500** | 5.95 | 2.42 | 10.31 | 7.13 | |
| **600** | 10.66 | 6.33 | 16.77 | 7.82 | |
| **700** | 20.71 | 9.15 | 20.55 | 9.83 | |
| **800** | 21.50 | 8.99 | 25.03 | 11.03 | |
| **900** | 25.30 | 11.36 | 28.81 | 11.64 | |

**Table S2** Summary of average reflectance of bare sapphire and various PdAuAg tri-metallic nanostructure annealed between 400 and 900 ^o^C.

| **Reflectance Summary [%]** | | |  |
| --- | --- | --- | --- |
| **Temperature [^o^C]** | **Tri-layer (15 nm)** | **Multi-layer (15 nm)** | |
| **Bare** | 7.97 | 7.97 | |
| **400** | 42.90 | 36.77 | |
| **500** | 40.76 | 31.61 | |
| **600** | 28.99 | 23.62 | |
| **700** | 22.20 | 15.04 | |
| **800** | 17.49 | 12.86 | |
| **900** | 15.21 | 11.23 | |

**Table S3** Raman summary of various PdAuAg tri-metallic nanostructures in terms of intensity, peak position of Raman band A1_g_.

| **(a)** | **Raman Summary (Tri-layer, 15 nm)** | |  |
| --- | --- | --- | --- |
| **Temperature [^o^C]** | **Intensity** | **Peak Position** | |
| **Bare** | 2525.31 | 417.91 | |
| **400** | 244.93 | 417.94 | |
| **500** | 241.61 | 417.96 | |
| **600** | 353.37 | 417.87 | |
| **700** | 626.95 | 417.81 | |
| **800** | 971.07 | 417.86 | |
| **900** | 1028.91 | 417.01 | |
| **(b)** | **Raman Summary (Multi-layer, 15 nm)** | |  |
| **Temperature [^o^C]** | **Intensity** | **Peak Position** | |
| **Bare** | 2525.31 | 417.91 | |
| **400** | 264.16 | 417.70 | |
| **500** | 271.59 | 417.83 | |
| **600** | 405.58 | 417.81 | |
| **700** | 792.95 | 417.88 | |
| **800** | 1010.85 | 417.84 | |
| **900** | 1190.37 | 417.73 | |

**Table S4** Summary of Rq, SAR and average reflectance of various PdAg nanostructures by the control annelaing duration with the Pd_150 nm_ / Ag_80 nm_ bilayers at 850 ^o^C on sapphire (0001) (Bi-layers).

| **Time [s]** | **Rq [nm]** | **SAR [%]** | **Reflectance [%]** |  |
| --- | --- | --- | --- | --- |
| **0** | 102.22 | 14.68 | 13.62 | |
| **240** | 162.79 | 14.27 | 2.82 | |
| **1800** | 161.38 | 14.11 | 3.67 | |
| **3600** | 125.93 | 10.12 | 2.83 | |
